# GenomeDepot: data management system for microbial comparative genomics

**DOI:** 10.1093/bioadv/vbag027

**Published:** 2026-01-29

**Authors:** Alexey Kazakov, Adam M Deutschbauer

**Affiliations:** Environmental Genomics and Systems Biology Division, Lawrence Berkeley National Laboratory, Berkeley, CA 94720, United States; Environmental Genomics and Systems Biology Division, Lawrence Berkeley National Laboratory, Berkeley, CA 94720, United States; Department of Plant and Microbial Biology, University of California, Berkeley, CA 94720, United States

## Abstract

**Summary:**

GenomeDepot is an open-source web-based platform for annotation, management, and comparative analysis of microbial genomic sequences and associated data including ortholog families, protein domains, operons, regulatory interactions, strain taxonomy, and sample metadata. GenomeDepot supports rapid creation of websites for user-defined genome collections that include bioinformatic tools for interactive genome browsing, Basic Local Alignment Search Tool (BLAST) search, annotation search, comparative genomic neighborhood visualization, and sequence download. Gene function annotations are generated by a customizable annotation pipeline. The pipeline runs annotation tools in Conda environments and can be easily extended with additional user-specified tools.

**Availability and implementation:**

GenomeDepot is open source and distributed under the GNU General Public License via GitHub (https://github.com/aekazakov/genome-depot). GenomeDepot is implemented in Python and was tested in Ubuntu Linux. Full installation instructions and documentation are available at https://aekazakov.github.io/genome-depot/. GenomeDepot demo server is freely accessible at https://iseq.lbl.gov/demogd/.

## 1 Introduction

Genome analysis tools play a crucial role in genomics research, enabling scientists to extract meaningful insights from genomic data. Comparative genomics serves as a powerful technique for functional annotation by leveraging evolutionary relationships and conservation patterns across species to assign biological functions to genes and genomic elements. Computational methods for functional gene annotation use homology search, domain and motif prediction, genomic context analysis, and statistical methods to transfer annotations from reference sequences and databases to the query sequences. Computational annotation tools vary in scope, focus, and level of detail in functional characterization of genes and genomic elements. General-purpose annotation tools, like Prokka (Seemann 2014), Bakta (Schwengers *et al.* 2021) or the Rapid Annotation of microbial genomes using Subsystems Technology (RAST) (Overbeek *et al.* 2014), are often integrated into larger pipelines with specialized annotation tools for in-depth analysis of genes related to particular functions or pathways (Dong and Strous 2019). Such pipelines are especially valuable for annotation of genomes of non-model organisms and understudied species. However, incompatible technical requirements, input file formats, and output data types hinder adding novel tools into existing computational pipelines. Furthermore, existing open-source platforms, while capable of exploring, searching, and visualizing multiple genomes (Carrere and Gouzy 2017, Holmer *et al*. 2019, Spoor *et al*. 2019, Fernandez-Pozo and Bombarely 2022, Roder *et al*. 2022, Marquis *et al*. 2024), often lack flexibility in configuration and data management or functionalities to unlock the full potential of comparative genomics analyses for investigation of gene function, regulation, and evolution.

To enable setting up interactive web portals for user-defined microbial genome collections we have developed GenomeDepot, an open-source web-based platform for management and analysis of microbial genomic sequences and associated data including ortholog families, protein domains, operons, regulatory interactions, metagenomic samples, and organism metadata. GenomeDepot includes an extensible genome annotation pipeline, relation database for data storage; and web-interface for data management, genome exploration, and comparative analysis.

## 2 Design and implementation

GenomeDepot is focused on genes as evolutionary and functional units. Many established algorithms for reconstruction of evolutionary relations (orthologs and paralogs) have quadratic time complexity because of the all-against-all search stage. For a genome database growing incrementally, orthology inference becomes more and more computationally expensive with each update. To avoid this pitfall, GenomeDepot uses precomputed ortholog groups (OGs) at several levels of taxonomy from the eggNOG database (Huerta-Cepas *et al*. 2019) for comparative analysis. Upon genome import, MD5 hash is generated for each protein sequence and compared with MD5 hashes of proteins in the database. Then, only novel protein sequences are mapped to eggNOG OGs by the eggNOG-mapper tool (Cantalapiedra *et al*. 2021). EggNOG-mapper aligns input proteins against a fixed set of reference sequences from the eggNOG database, thus ensuring nearly linear time complexity at any genome database size.

For functional characterization of protein-coding genes, eggNOG-mapper provides function annotations from several classifications like Kyoto Encyclopedia of Genes and Genomes (KEGG) orthologs, reactions and pathways, EC numbers, and Gene Ontology labels. In addition to genes, GenomeDepot database can store information about other genomic features, such as predicted operons, regulatory interactions (regulons), and transcription factor binding sites.

**Figure 1:**
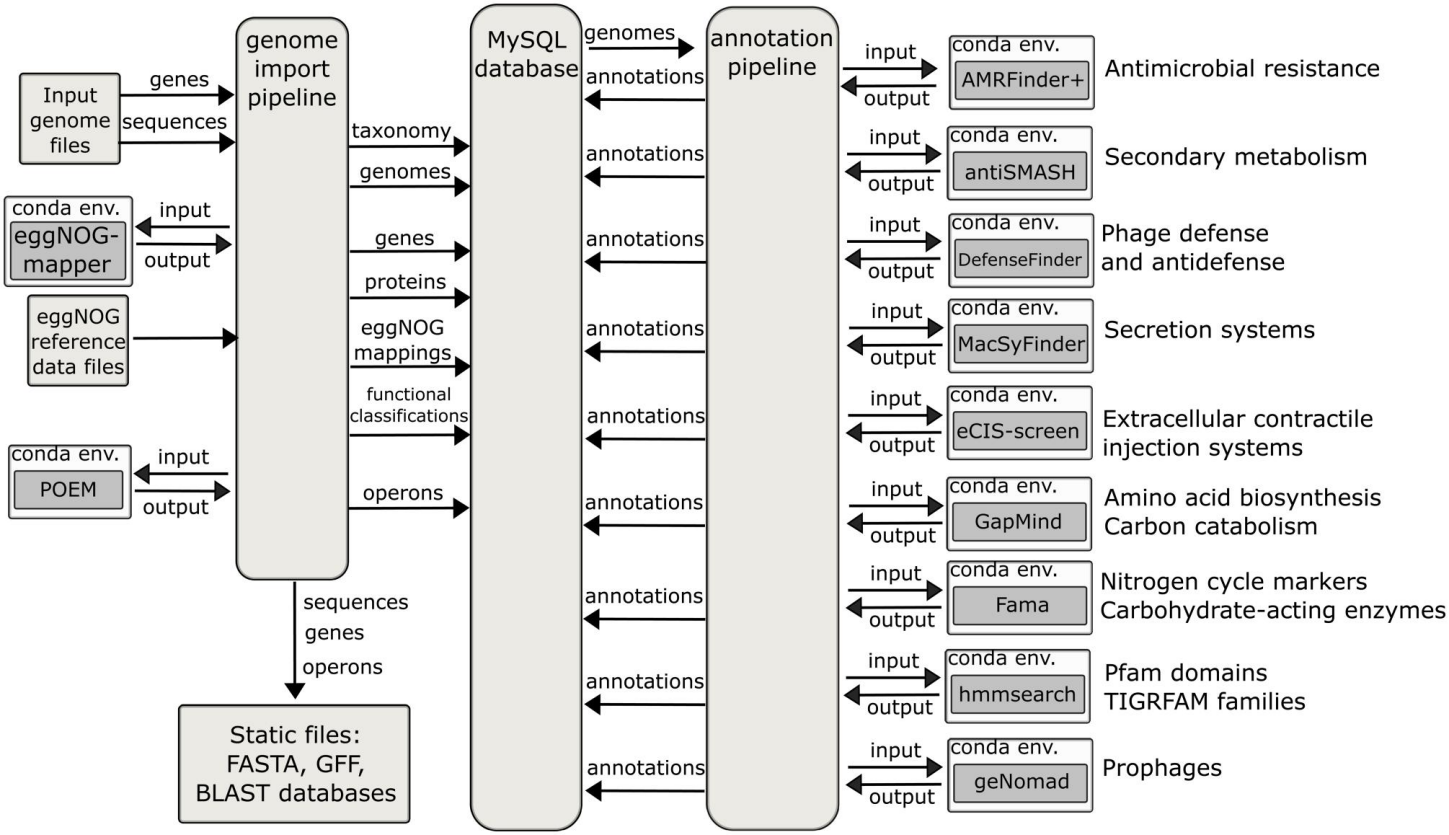
Data generation pipelines of GenomeDepot. Computational tools supported by the annotation pipeline are listed in File 1, available as supplementary data at *Bioinformatics Advances* online.

Additional annotations of gene functions are generated by the GenomeDepot annotation pipeline (Fig. 1). The annotation pipeline runs an array of functional annotation tools. To satisfy system requirements and avoid software conflicts between annotation tools, each tool is installed in an individual Conda virtual environment and wrapped into a bash script. The annotation pipeline generates gene annotations by calling Python-based plugins. A plugin creates input files for the annotation tool, generates and runs a bash script invoking the tool, extracts gene annotations from the tool’s output files and creates a tab-separated text file with annotations for import into the database. Then, the annotation pipeline reads the tab-separated file and writes gene function annotations into the database. Currently, the annotation pipeline supports nine annotation tools listed in File 1, available as supplementary data at *Bioinformatics Advances* online, but it can be easily extended with additional plugins for more tools. In addition, gene annotations generated outside of GenomeDepot pipelines can be imported into the database from tab-separated text files.

For the data generation, the genome import pipeline of GenomeDepot extracts sequences and annotated genes from input GenBank-formatted files and associates taxonomic information in the files with NCBI taxonomy identifiers. Next, it runs eggNOG-mapper, collects mappings of proteins to functional classifications from the eggNOG-mapper output and populates MySQL database with gene entries, protein sequences, and protein mappings. Then, the pipeline runs Pipeline for Operon Exploration in Metagenomes (POEM) to predict operons and saves operon entries in the database. Finally, it generates nucleotide FASTA and General Feature Format (GFF) files for the JBrowse genome viewer and updates nucleotide and protein BLAST databases. After genome import, the annotation pipeline sequentially runs genome analysis tools for each new genome, reads their output and writes gene annotations to the database. Scheduling and executing pipeline jobs are handled by Django Q, a multiprocessing task queue.

**Figure 2:**
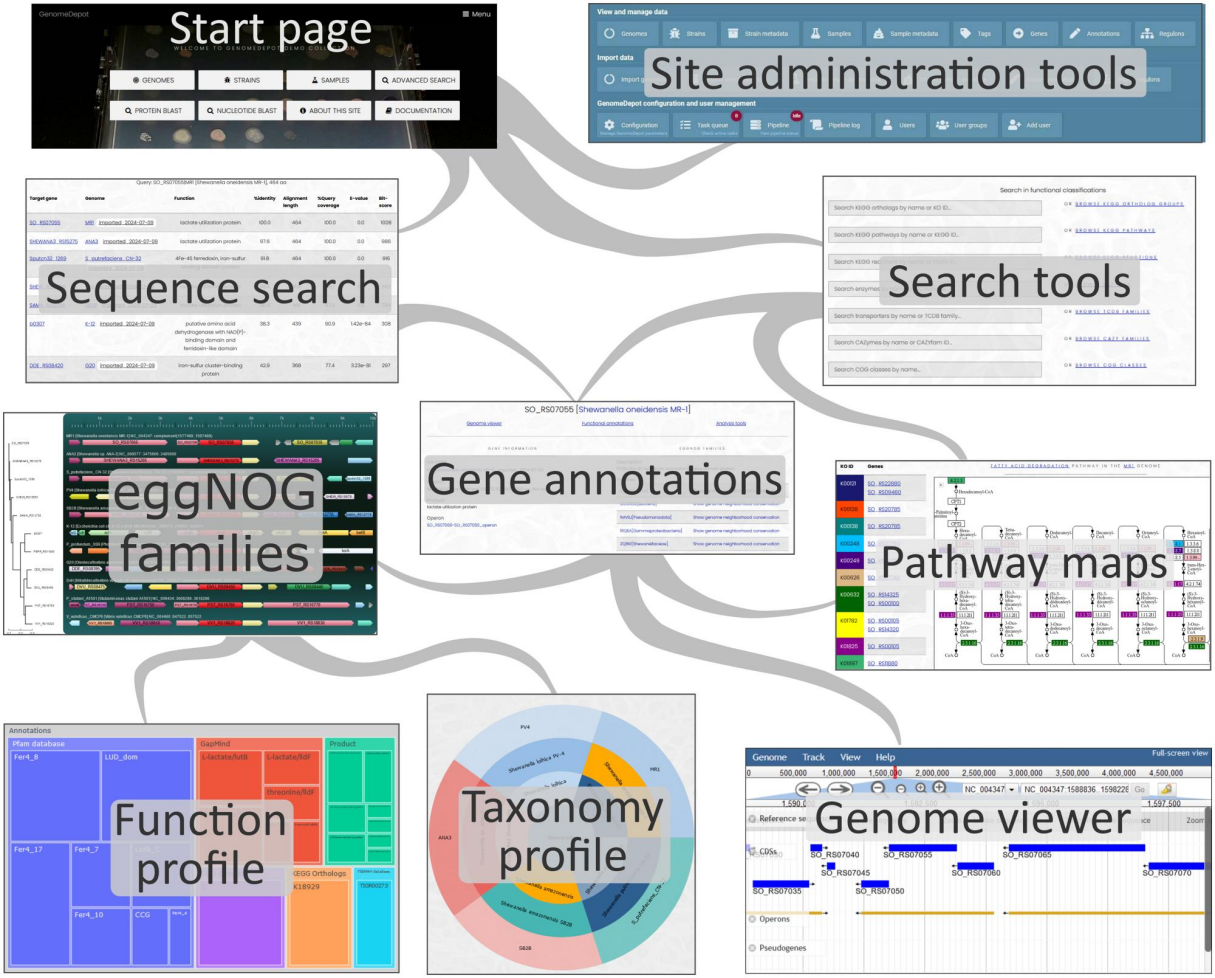
Overview of GenomeDepot key capabilities.

An overview of a GenomeDepot-powered website capabilities is shown in Fig. 2. All the data are stored in a relational MySQL database, except nucleotide sequences, which are stored as compressed FASTA- and GenBank-formatted files. GenomeDepot web application executes MySQL database queries and delivers front-end visualization using Django, a high-level Python web framework (https://www.djangoproject.com/). Front-end user interface uses CSS and JavaScript; and incorporates JBrowse genome browser for interactive genome visualization. GenomeDepot has Python-based web applications for nucleotide and protein sequence search with BLAST, comparative analysis of genome neighborhood, conserved operons, and conserved regulons. These applications run asynchronously to avoid front-end web page loading delay. In addition, Django provides a built-in user authentication module and web-site administration panel.

By default, multiple installations of GenomeDepot on one server share a single Conda environment for each annotation tool. If a tool would be installed in several Conda environments (for example, to support different versions of the tool), a specific name of the environment can be specified in the admin web-interface of a GenomeDepot installation. In addition, configuration parameters for annotation tools can be set in the admin web-interface and stored in the database.

GenomeDepot permits flexible organization of microbial genome collections. GenomeDepot can import genome files in GenBank format generated by Prokka (Seemann 2014), Bakta (Schwengers *et al*. 2021) or similar tools, as well as genome assemblies downloaded from NCBI Datasets (O’Leary *et al*. 2024) or DOE KBase (Arkin *et al.* 2018). GenomeDepot supports multiple installations in one host system to store the data in separate databases, while sharing the Conda environments and annotation tools to minimize storage space overhead. Thus, genomes from several projects can be hosted in one database or in separate databases, with separate access permissions. For example, project administrators can restrict access to a collection of pre-publication data and keep public genomes in a separate database open to the public. Alternatively, genomes from separate projects can be stored independently and accessed by different groups of users.

### 2.1 GenomeDepot features

Genomic data in GenomeDepot is presented in the form of pages containing either a list of entries or a detailed view of an entry. GenomeDepot provides detailed view pages for genomes, strains (source organisms of the genomes), samples (sources of metagenome-assembled genomes), taxa, and genome features like genes, operons, binding sites, and regulons. The pages for genomes and genome features display the interactive genome browser JBrowse. In addition, the gene detail page contains links to a comparative gene neighborhood application and to external tools for genome analysis.

The page for gene conserved neighborhood analysis displays a region around a selected gene and its orthologs from a selected eggNOG family. This application finds 10–200 proteins most similar to the starting protein, arranges them according to the phylogenetic tree and displays neighboring genes colored by the eggNOG family. The page also shows a treemap chart with a functional profile of all genes in the neighborhood.

The page with a list of genomes contains a hierarchical interactive sunburst plot displaying a taxonomic overview of the genome collection. A click on a sector zooms in/out on the plot, while a click on a taxon or genome name opens a details page for the taxon or genome.

GenomeDepot provides several tools for searching through genome dataset. On the search page, users can search for genes by gene name, by annotated function, or by one of functional classifications like KEGG orthologs, KEGG pathways, and Gene Ontology terms across all genomes. Similarly, on the genome detail page users can search in gene annotations and functional classifications for the current genome only. Sequence similarity searches can be performed through pages for nucleotide (megaBLAST) or protein (BLASTp) sequence search. Users can also identify genome or strain of interest by searching in genome names, strain names, taxonomy, or strain metadata entries.

From the genome details page, users can open a list of KEGG pathways identified in the genome, then click on a pathway ID to display a list of genes mapped to the pathway. A link at the top of the gene list opens a webpage with a table of KEGG orthology groups and genes from that pathway and a panel with a pathway map with matching colored marks from the KEGG website.

### 2.2 Website administration

In addition to the pipeline-generated data, administrators of a GenomeDepot-based portal can use the web-based administration panel and the command-line utility to add, change, or delete database content. The GenomeDepot administration panel provides access to individual data entries and GenomeDepot tools. The GenomeDepot Tools page of the administration panel provides access to bulk import tools for genomes, strain metadata, sample descriptions, sample metadata, gene annotations, and regulons. A comprehensive description of the administration panel and command-line utility is available in the GenomeDepot documentation.

## 3 Conclusion

In this work, we report a new software/database system for microbial genomic data management and comparative analysis. With GenomeDepot, research teams can organize their genome collections, combine them with genomes downloaded from public sources, run an array of annotation tools, and present them in web-portals.

We benchmarked the time of genome import and annotation by running GenomeDepot on two computational platforms: workstation (Intel Xeon 6-core 2.4Ghz, 64 Gb RAM) and server (AMD EPYC 32-core 2.7Ghz, 512 Gb RAM). Two testing datasets (30 and 100 genomes) were imported into an empty database. In addition, we tested an update of the 100-genome database with 100 unrelated bacterial genomes to estimate the change of the runtime with the increase of the database size. For the workstation, genome import took 8 minutes/genome for the 30-genomes dataset and 5 minutes/genome for both 100-genomes datasets. For the server, genome import took 2–3 minutes/genome, with the smallest dataset demonstrating the lowest processing speed. It is worth noting that running eggNOG-mapper and POEM took 85%–90% of the total genome import time. The processing time of the annotation pipeline was 9.5–11 minutes/genome for the workstation and 5–6 minutes/genome for the server. Full description of the benchmarking results is available in File 1, available as supplementary data at *Bioinformatics Advances* online.

To determine minimal CPU and memory requirements, we installed GenomeDepot in an Oracle VirtualBox machine running Ubuntu 22.04 server. The genome import and genome annotation pipelines successfully processed a 30-genomes dataset with 8 Gb RAM and 4 virtual CPUs allocated to the virtual machine. The limiting factor was the high memory requirement of eggNOG-mapper. After importing the genomes, GenomeDepot web application can work with as low as 2 Gb RAM and 2 CPUs allocated.

For the demonstration of GenomeDepot capabilities, we set up a web portal with 30 bacterial genomes, freely accessible at https://iseq.lbl.gov/demogd/. We also tested GenomeDepot usability in a larger dataset of 2205 bacterial and archaeal genomes, accessible for the general public at https://iseq.lbl.gov/genomes/.

## Supplementary Material

vbag027_Supplementary_Data

## Data Availability

GenomeDepot source code is available on GitHub at https://github.com/aekazakov/genome-depot/. Documentation is available online at https://aekazakov.github.io/genome-depot/.

## Supplementary material

A GenomeDepot demonstration version installed in Ubuntu Server is available for download as a virtual machine image for VirtualBox 7 at https://doi.org/10.5281/zenodo.17917445
