# Supplementary material for: GenomeDepot: data management system for microbial comparative genomics
Source: Bioinform Adv. 2026 Jan 29;6(1):vbag027. doi: 10.1093/bioadv/vbag027 (PMC12895066; doi:10.1093/bioadv/vbag027)
Supplement: vbag027_Supplementary_Data [file vbag027_supplementary_data.pdf]

1. Gene function annotation tools included into GenomeDepot annotation pipeline

AMRFinderPlus https://github.com/ncbi/amr

Identification of antimicrobial resistance genes

License: Public Domain

Reference: Feldgarden M, Brover V, Gonzalez-Escalona N, Frye JG, Haendiges J, Haft DH, Hoffmann M, Pettengill JB, Prasad AB, Tillman GE, Tyson GH, Klimke W. AMRFinderPlus and the Reference Gene Catalog facilitate examination of the genomic links among antimicrobial resistance, stress response, and virulence. Sci Rep. 2021 Jun 16;11(1):12728. doi: 10.1038/s41598-021-91456-0.

antiSMASH https://github.com/antismash/antismash

Identification of secondary metabolite biosynthesis genes

License: GNU GPL v3.0

Reference: Blin K, Shaw S, Kloosterman AM, et al. antiSMASH 6.0: improving cluster detection and comparison capabilities. Nucleic Acids Res. 2021;49(W1):W29-W35. doi:10.1093/nar/gkab335.

eCIS-screen https://github.com/ipb-jianyang/eCIS-screen

Identification of extracellular Contractile Injection System (eCIS) genes

License: GPL-3.0 license

Reference: Chen L, Song N, Liu B, Zhang N, Alikhan NF, Zhou Z, Zhou Y, Zhou S, Zheng D, Chen M, Hapeshi A, Healey J, Waterfield NR, Yang J, Yang G. Genome-wide Identification and Characterization of a Superfamily of Bacterial Extracellular Contractile Injection Systems. Cell Rep. 2019 Oct 8;29(2):511-521.e2. doi: 10.1016/j.celrep.2019.08.096.

Fama https://github.com/aekazakov/Fama

Identification of nitrogen cycle markers, carbohydrate-acting enzymes and universal marker genes

License: LBNL BSD 3-clause

Reference: Kazakov A, Novichkov P. Fama: a computational tool for comparative analysis of shotgun metagenomic data. Great Lakes Bioinformatics conference (poster presentation). 2019. https://iseq.lbl.gov/mydocs/fama_glbio2019_poster.pdf

GapMind https://github.com/morgannprice/PaperBLAST

Identification of amino acid biosynthesis and carbon catabolism genes

License: GPL-3.0 license

Reference: Price MN, Deutschbauer AM, Arkin AP. GapMind: Automated Annotation of Amino Acid Biosynthesis. mSystems. 2020 Jun 23;5(3):e00291-20. doi: 10.1128/mSystems.00291-20.

DefenseFinder https://github.com/mdmparis/defense-finder

Identification of phage defense and anti-defense genes

License: GPL-3.0 license

Reference: Tesson F, Hervé A, Mordret E, et al. Systematic and quantitative view of the antiviral arsenal of prokaryotes. Nat Commun. 2022;13(1):2561. doi:10.1038/s41467-022-30269-9

MacSyFinder https://github.com/gem-pasteur/macsyfinder

Identification of bacterial secretion and type IV-filament genes

License: GPL-3.0 license

Reference: Abby SS, Néron B, Ménager H, Touchon M, Rocha EP. MacSyFinder: a program to mine genomes for molecular systems with an application to CRISPR-Cas systems. *PLoS One*. 2014;9(10):e110726. doi:10.1371/journal.pone.0110726

geNomad https://github.com/apcamargo/genomad

Identification of prophage genes

License: ACADEMIC, INTERNAL, RESEARCH & DEVELOPMENT, NON-COMMERCIAL USE ONLY, LICENSE

Reference: Camargo, A.P., Roux, S., Schulz, F. et al. Identification of mobile genetic elements with geNomad. Nat Biotechnol 42, 1303–1312 (2024). doi:10.1038/s41587-023-01953-y

HMMER https://github.com/EddyRivasLab/hmmer

Identification of protein domains (Pfam) and mapping of protein families (TIGRFAM)

License: BSD 3-clause

References:

1. Eddy SR. Accelerated Profile HMM Searches. PLoS Comput Biol. 2011;7(10):e1002195. doi:10.1371/journal.pcbi.1002195
2. Haft DH, Selengut JD, White O. The TIGRFAMs database of protein families. Nucleic Acids Res. 2003;31(1):371-373. doi:10.1093/nar/gkg128
3. Mistry J, Chuguransky S, Williams L, et al. Pfam: The protein families database in 2021. Nucleic Acids Res. 2021;49(D1):D412-D419. doi:10.1093/nar/gkaa913

2. Genome import benchmarking

We benchmarked runtime of data generation by GenomeDepot pipelines on two computational platforms, a workstation and a rack-mounted server. The tested scenarios included creation of small-size (30 genomes), medium-size (100 genomes), and large-size (1000 genomes) genome collections, as well as adding 100 genomes to an existing database. To minimize the number of redundant proteins, we selected genomes from different species for medium-size and large-size datasets. The running time of all steps of genome import and genome annotation pipelines is shown in the table 1. The creation of 1000-genome dataset on the workstation would take more than 9 days, so we excluded this test from the benchmarking. The processing rate of genome import varied from 2 minutes/genome for the largest dataset on the server to 8 minutes/genome for the smallest dataset on the workstation. During the genome import, mapping of proteins to eggNOG famileis with eggNOG-mapper and predicting operons with POEM were the most computation-heavy steps, which together took 85-90% of the total genome import time. Since the genome import pipeline combines proteins from all genomes into a single input file for eggNOG-mapper, genome import of small-size batches is less efficient than importing one large batch. However, importing of a large number of genomes in one batch requires tuning of MySQL settings (InnoDB buffer pool size, in particular). The processing time of the annotation pipeline was 9.5-11 minutes/genome for the workstation and 5-6 minutes/genome for the server. In general, increasing the number of available hardware threads decreases the runtime of the GenomeDepot annotation pipeline. However, some tools in the pipeline use parallelization less efficient than the others. Thus, in our tests, x4 increase of the number of threads resulted only in x2 decrease of the annotation pipeline runtime.

2.1 Testing platforms

- Workstation.
    - Hardware: Intel Xeon 6-core 2.4 Ghz CPU (12 hardware threads), 64 Gb RAM, 1.8 Tb HDD storage
    - Software configuration: Ubuntu 22.04 OS, 8 threads available for GenomeDepot tools, InnoDB buffer pool size 4 Gb
- Server.
    - Hardware: AMD EPYC 32-core 2.5 Ghz CPU (64 hardware threads), 512 Gb RAM, 17 Tb RAID5 storage

- Software configuration: Ubuntu Server 22.04 OS, 32 threads available for GenomeDepot tools, InnoDB buffer pool size 192 Gb

Table 1. Benchmark of GenomeDepot data generation. The time is shown in hours and minutes.

Testing platform	Workstation			Server			
Dataset size, genomes	30	100	Add 100	30	100	Add 100	1000
**Genome import pipeline, total time**	**3:59**	**8:35**	**8:22**	**1:17**	**3:19**	**3:02**	**31:06**
Input files processing	0:02	0:05	0:06	0:01	0:04	0:05	0:38
EggNOG-mapper runtime	2:34	4:56	5:04	0:31	0:58	0:54	7:58
EggNOG-mapper output processing	<0:01	<0:01	<0:01	<0:01	<0:01	<0:01	0:02
MySQL database update	0:18	0:12	0:17	0:04	0:07	0:10	1:17
POEM runtime	0:56	2:53	2:29	0:33	1:49	1:34	17:42
Operon predictions import	0:01	0:01	0:01	0:01	0:01	0:01	0:12
Static files generation	0:08	0:28	0:23	0:06	0:20	0:17	3:18
**Annotation pipeline, total time**	**5:43**	**16:44**	**15:40**	**3:05**	**9:28**	**8:35**	**90:10**
AMRfinder+	0:05	0:16	0:14	0:02	0:05	0:05	0:49
antiSMASH	0:23	1:00	1:03	0:22	1:02	0:59	12:40
DefenseFinder	0:09	0:30	0:26	0:04	0:12	0:11	1:54
eCIS_screen	0:02	0:05	0:04	0:05	0:18	0:13	2:36
Fama	0:33	1:09	1:03	0:02	0:04	0:04	0:30
Gapmind	0:17	0:53	0:54	0:06	0:18	0:18	2:55
geNomad	1:58	6:08	5:59	0:46	2:12	2:12	21:40
hmmsearch/ Pfam	1:36	4:51	4:14	1:12	3:56	3:23	**34:49**
hmmsearch/ TIGRFAM	0:28	1:17	1:11	0:17	0:52	0:45	7:50
MacSyFinder	0:12	0:35	0:32	0:09	0:28	0:25	4:27

2.2 Test datasets

- 30 genomes of Bacteria (same as in https://iseq.lbl.gov/demogd/) imported into an empty database.
- 100 genomes of Bacteroidota imported into an empty database, no more than one genome per species, average genome size 4.7 Mbp (table 2).
- 100 genomes from 20 bacterial phyla imported into the database of 100 Bacteroidota genomes. All genomes from different species, average genome size 3.8 Mbp (table 3).
- 1000 genomes of Archaea and Bacteria imported into an empty database, no more than one genome per species, average genome size 3.9 Mbp (table 4).

All genome assemblies have been downloaded from NCBI before testing.

Table 2. Test dataset for generation of database of 100 genomes

Genome	Size, bp	NCBI accession number
Bacteroides cellulosilyticus DSM 14838	6870144	NZ_ACCH01000000
Bacteroides coprosuis DSM 18011	2991798	NZ_AFFW01000000
Bacteroides eggerthii DSM 20697	4197635	NZ_ABVO01000000
Bacteroides finegoldii CL09T03C10	5124109	NZ_AKBZ01000000
Bacteroides helcogenes P 36-108	3998906	NC_014933
Bacteroides sp. 2_2_4	7101224	NZ_ABZZ01000000
Bacteroides thetaiotaomicron VPI-5482	6293399	NC_004703
Bacteroides uniformis ATCC 8492	4719097	NZ_AAYH02000000
Phocaeicola coprophilus DSM 18228 = JCM 13818	4041504	NZ_ACBW01000000
Phocaeicola dorei 5_1_36/D4	5538248	NZ_ACDI02000000
Phocaeicola vulgatus ATCC 8482	5163189	NC_009614
Paludibacter propionicigenes WB4	3685504	NC_014734
Bacteroides dorei CL03T12C01	5310365	NZ_CP011531
Bacteroides vulgatus CL09T03C04	4916502	NZ_AGXZ01000000
Porphyromonas asaccharolytica DSM 20707	2186370	NC_015501
Prevotella copri DSM 18205	3512473	NZ_ACBX02000000
Prevotella ruminicola 23	3619559	NC_014033
Alistipes finegoldii DSM 17242	3734239	NC_018011
Rikenella microfusus DSM 15922	2880076	NZ_ATXW01000000
Parabacteroides sp. 2_1_7	5166302	NZ_ABZY02000000
Chitinophaga pinensis DSM 2588	9127347	NZ_ABTK01000000
Niabella soli DSM 19437	4697343	NZ_AGSA01000000
Niastella koreensis GR20-10	9033684	NC_016609
Bernardetia litoralis DSM 6794	4919337	NC_018018
Belliella baltica DSM 15883	4196595	NC_018010
Echinicola vietnamensis DSM 17526	5608040	NC_019904
Flectobacillus major DSM 103	6221030	NZ_ATXY01000000

Spirosoma linguale DSM 74	8491258	ABUR01000000
Pontibacter actiniarum	4974797	NZ_CP021236
Fluviicola taffensis DSM 16823	4633577	NC_015321
Aequorivita sublithincola DSM 14238	3520671	NC_018013
Cellulophaga lytica DSM 7489	3765936	NC_015167
Gillisia limnaea DSM 15749	3966857	NZ_AHKR01000000
Joostella marina DSM 19592	4508243	NZ_AJUG01000000
Muricauda ruestringensis DSM 13258	3842422	NC_015945
Psychroflexus torquis ATCC 700755	4321832	NC_018721
Polaribacter irgensii 23-P	2763458	NZ_AAOG01000000
Polaribacter sp. MED152	2961474	NC_020830
Ornithobacterium rhinotracheale DSM 15997	2399175	NC_018016
Riemerella anatipestifer ATCC 11845 = DSM 15868	2155121	NC_014738
Flavobacteria bacterium BAL38	2806989	AAXX01000000
Flavobacteriales bacterium ALC-1	3825707	ABHI01000000
Flavobacteria bacterium BBFL7	3103424	AAPD01000000
Haliscomenobacter hydrossis DSM 1100	8771651	NC_015513
Pedobacter heparinus DSM 2366	5167383	NZ_ABUJ01000000
Pedobacter sp. BAL39	5788054	NZ_ABCM01000000
Pseudopedobacter saltans DSM 12145	4635236	NZ_AEKX01000000
Sphingobacterium spiritivorum ATCC 33861	5093512	NZ_ACHA02000000
Sphingobacteriaceae bacterium GW460-11-11-14-LB5	6182066	NZ_CP021237
Rhodothermus marinus DSM 4252	3386737	NZ_ABUL01000000
Salinibacter ruber DSM 13855	3587328	NC_007678
Bacteroides finegoldii DSM 17565	4892401	NZ_ABXI02000000
Bacteroides fragilis 3_1_12	5530115	NZ_ABZX01000000
Bacteroides intestinalis DSM 17393	6052596	NZ_ABJL02000000
Bacteroides ovatus ATCC 8483	6465369	NZ_AAXF02000000
Bacteroides sp. 4_3_47FAA	5442925	ACDR02000000
Bacteroides sp. 9_1_42FAA	5618568	ACAA02000000
Bacteroides sp. D1	5974559	NZ_ACAB02000000
Bacteroides stercoris ATCC 43183	4009829	NZ_ABFZ02000000
Phocaeicola coprocola DSM 17136	4300117	NZ_ABIY02000000
Phocaeicola dorei DSM 17855	5566217	NZ_ABWZ01000000
Phocaeicola plebeius DSM 17135	4421924	NZ_ABQC02000000
Phocaeicola salanitronis DSM 18170	4308663	NC_015166
Barnesiella viscericola DSM 18177	3076856	NZ_CP007034
Odoribacter splanchnicus DSM 20712	4392288	NC_015160
Porphyromonas gingivalis ATCC 33277	2354886	NC_010729
Prevotella bivia DSM 20514	2521238	NZ_AJVZ01000000
Prevotella multisaccharivorax DSM 17128	3388644	NZ_AFJE01000000

Alistipes putredinis DSM 17216	2550678	NZ_ABFK02000000
Parabacteroides distasonis ATCC 8503	4811379	NC_009615
Parabacteroides johnsonii DSM 18315	4787097	NZ_ABYH01000000
Parabacteroides merdae ATCC 43184	4434377	NZ_AAXE02000000
Saccharicrinis fermentans DSM 9555 = JCM 21142	5982061	NZ_AZYH01000000
Candidatus Amoebophilus asiaticus 5a2	1884364	NZ_ABLI01000000
Algoriphagus machipongonensis	4787724	NZ_AAXU02000000
Aquiflexum balticum DSM 16537	5987195	NZ_LT838813
Cyclobacterium marinum DSM 745	6221273	NC_015914
Cytophaga hutchinsonii ATCC 33406	4433218	NZ_AABD03000000
Hymenobacter roseosalivarius DSM 11622	4948485	NZ_FWWW01000000
Marivirga tractuosa DSM 4126	4516490	NC_014750
Microscilla marina ATCC 23134	9771226	NZ_AAWS01000000
Dyadobacter fermentans DSM 18053	6967790	NZ_ABTS01000000
Leadbetterella byssophila DSM 17132	4059653	NC_014655
Runella slithyformis DSM 19594	6919729	NC_015695
Capnocytophaga ochracea DSM 7271	2612925	NZ_ABTH01000000
Capnocytophaga sputigena ATCC 33612	2997845	NZ_ABZV01000000
Cellulophaga algicola DSM 14237	4888353	NC_014934
Croceibacter atlanticus HTCC2559	2952962	NC_014230
Dokdonia sp. MED134	3302548	NZ_CP009301
Flavobacterium johnsoniae UW101	6096872	NZ_AAPM01000000
Flavobacterium psychrophilum JIP02/86	2860382	NC_009613
Gramella forsetii KT0803	3798465	NC_008571
Kordia algicida OT-1	5033129	NZ_ABIB01000000
Leeuwenhoekiella blandensis MED217	4244565	NZ_AANC01000000
Maribacter sp. HTCC2170	3868304	NC_014472
Robiginitalea biformata HTCC2501	3530383	NC_013222
Zunongwangia profunda SM-A87	5128187	NC_014041
Flavobacteriaceae bacterium 3519-10	2768102	CP001673
Mucilaginibacter paludis DSM 18603	8408322	NZ_AEIH02000000
Sphingobacterium spiritivorum ATCC 33300	5232948	NZ_ACHB01000000

Table 3. Test dataset for update of a 100-genomes database with 100 unrelated genomes

Genome	Size, bp	NCBI accession number
Acidobacterium capsulatum ATCC 51196	4127356	NC_012483
Candidatus Solibacter usitatus Ellin6076	9965640	AAIA01000000
Acidimicrobium ferrooxidans DSM 10331	2158157	NZ_ABSZ01000000
Acidothermus cellulolyticus 11B	2443540	NZ_AAOL01000000
Gardnerella vaginalis 409-05	1617545	NC_013721
Actinospica robiniae DSM 44927	9918887	NZ_AZAN01000000

Catenulispora acidiphila DSM 44928	10467782	NZ_ABTI01000000
Lancefieldella parvulum DSM 20469	1543805	NZ_ABTD01000000
Slackia heliotrinireducens DSM 20476	3165038	NZ_ABUP01000000
Mycobacterium avium 104	5475491	NC_008595
Frankia sp. EAN1pec	8982042	AAII01000000
Geodermatophilus obscurus DSM 43160	5322497	NZ_ABTU01000000
Stackebrandtia nassauensis DSM 44728	6841557	NZ_ABUS01000000
Beutenbergia cavernae DSM 12333	4669183	NZ_ABTE01000000
Brachybacterium faecium DSM 4810	3614992	NZ_ABTF01000000
Dermacoccus sp. SAI-028	3141763	NZ_SMFS01000000
Intrasporangium calvum	4025044	NZ_CP031145
Jonesia denitrificans DSM 20603	2749646	NZ_ABUA01000000
Kytococcus sedentarius DSM 20547	2785024	NZ_ABUD01000000
Rothia mucilaginosa DY-18	2264603	NC_013715
Sanguibacter keddieii DSM 10542	4253413	NZ_ABUN01000000
Nakamurella multipartita DSM 44233	6060298	NZ_ABUH01000000
Kribbella flavida DSM 17836	7579488	NZ_ABUC01000000
Cutibacterium acnes KPA171202	2560265	NC_006085
Rubrobacter xylanophilus DSM 9941	3225748	NZ_AAEB02000000
Thermobifida fusca YX	3642249	AAAQ02000000
Thermomonospora curvata DSM 43183	5639016	NZ_ABUZ01000000
Conexibacter woesei DSM 14684	6359369	NZ_ABTL01000000
Sulfurihydrogenibium azorense Az-Fu1	1640877	NC_012438
Desulfurobacterium thermolithotrophum DSM 11699	1541968	NC_015185
Caldithrix abyssi DSM 13497	4955439	NZ_AGCQ01000000
Candidatus Cloacimonas acidaminovorans str. Evry	2246820	NC_020449
Candidatus Protochlamydia amoebophila UWE25	2438912	NC_005861
Chlorobaculum parvum NCIB 8327	2289249	NC_011027
Roseiflexus sp. RS-1	5801598	NZ_AAQU01000000
Dehalococcoides mccartyi 195	1469720	NC_002936
Coprothermobacter proteolyticus DSM 5265	1424912	NC_011295
Gloeobacter violaceus PCC 7421	4659019	NC_005125
Microcystis aeruginosa NIES-843	5842795	NC_010296
Thermosynechococcus vestitus BP-1	2593857	NC_004113
Parasynechococcus marenigrum WH 8102	2434428	AABA02000000
Flexistipes sinusarabici DSM 4947	2526590	NC_015672
Denitrovibrio acetiphilus DSM 12809	3222077	NZ_ABTN01000000
Dictyoglomus thermophilum H-6-12	1959987	NC_011297
Elusimicrobium minutum Pei191	1643562	NZ_ABIQ01000000
Fibrobacter succinogenes subsp. succinogenes S85	3842635	NZ_ACRA01000000
Streptococcus uberis 0140J	1852352	NC_012004

Alkaliphilus metalliredigens QYMF	4929566	NZ_AAKU01000000
Halobacteroides halobius DSM 5150	2649255	NC_019978
Caldanaerobacter subterraneus subsp. tengcongensis MB4	2689445	NC_003869
Acidaminococcus fermentans DSM 20731	2329769	NC_013740
Veillonella parvula DSM 2008	2132142	NZ_ABVB01000000
Leptotrichia buccalis C-1013-b	2465610	NZ_ABUE01000000
Gemmatimonas aurantiaca T-27	4636964	NC_012489
Thermodesulfovibrio yellowstonii DSM 11347	2003803	NC_011296
Rubinisphaera brasiliensis DSM 5305	6006602	NZ_AEIC01000000
Acidithiobacillus ferrooxidans ATCC 53993	2885038	NC_011206
Brevundimonas sp. GW460-12-10-14-LB2	3263775	NZ_CP015511
Magnetococcus marinus MC-1	4719581	NZ_AAAN03000000
Maricaulis maris MCS10	3368780	NZ_AASK01000000
Parvularcula bermudensis HTCC2503	2902643	NZ_CH724133
Candidatus Pelagibacter ubique HTCC1062	1308759	NC_007205
Erythrobacter litoralis HTCC2594	3052398	NZ_CM000156
Chromobacterium violaceum ATCC 12472	4751080	NC_005085
Thiobacillus denitrificans ATCC 25259	2909809	NZ_AAFH01000000
Dechloromonas aromatica RCB	4501104	AADF01000000
Desulfococcus oleovorans Hxd3	3944167	NZ_AAWN01000000
Hippea maritima DSM 10411	1694430	NC_015318
Desulfobacca acetoxidans DSM 11109	3282536	NC_015388
Syntrophobacter fumaroxidans MPOB	4990251	NZ_AAJF01000000
Aliarcobacter butzleri RM4018	2341251	NC_009850
Nitratiruptor sp. SB155-2	1877931	NC_009662
Aeromonas hydrophila subsp. hydrophila ATCC 7966	4744448	NC_008570
Tolumonas auensis DSM 9187	3471292	NZ_ACFZ01000000
Dichelobacter nodosus VCS1703A	1389350	NC_009446
Cellvibrio japonicus Ueda107	4576573	NC_010995
Halothiobacillus neapolitanus c2	2582886	NZ_ACJO01000000
Citrobacter portucalensis	4945242	NZ_CP022311
Kangiella koreensis DSM 16069	2852073	NZ_ABUB01000000
Coxiella burnetii CbuG_Q212	2008870	NC_011527
Methylococcus capsulatus str. Bath	3304561	NC_002977
Marinomonas sp. MWYL1	5100344	AAVH01000000
Actinobacillus succinogenes 130Z	2319663	AAKC01000000
Pseudomonas aeruginosa PAO1	6264404	NC_002516
Francisella tularensis subsp. mediasiatica FSC147	1893886	CP000915
Stenotrophomonas maltophilia K279a	4851126	NC_010943
Bdellovibrio bacteriovorus HD100	3782950	NC_005363
Gordonia bronchialis DSM 43247	5290012	NZ_ABTV01000000

Xylanimonas cellulosilytica DSM 15894	3831380	NZ_ABVC01000000
Nocardioides sp. JS614	5293685	NZ_AAJB01000000
Streptosporangium roseum DSM 43021	10369518	NZ_ABUU01000000
Aquifex aeolicus VF5	1590791	NC_001880
Sphaerobacter thermophilus DSM 20745	3993764	NZ_ABUQ01000000
Thermobaculum terrenum ATCC BAA-798	3101581	NZ_ABUX01000000
Thermomicrobium roseum DSM 5159	2920744	NC_011961
Calditerrivibrio nitroreducens DSM 19672	2216552	NC_014749
Deferribacter desulfuricans SSM1	2542933	NC_013940
Thermus thermophilus HB27	2127482	NC_005838
Isosphaera pallida ATCC 43644	5529304	NC_014957
Xanthobacter autotrophicus Py2	5625098	AAPC01000000

Table 4. Test dataset for generation of database of 1000 genomes

Genome	Size, bp	NCBI accession number
Acidobacterium capsulatum ATCC 51196	4127356	NC_012483
Candidatus Koribacter versatilis Ellin345	5650368	NC_008009
Candidatus Solibacter usitatus Ellin6076	9965640	AAIA01000000
Acidimicrobium ferrooxidans DSM 10331	2158157	NZ_ABSZ01000000
Acidothermus cellulolyticus 11B	2443540	NZ_AAOL01000000
Actinomyces urogenitalis DSM 15434	2702812	NZ_ACFH01000000
Fannyhessea vaginae DSM 15829	1418601	NZ_ADNA01000000
Gleimia coleocanis DSM 15436	1723843	NZ_ACFG01000000
Mobiluncus curtisii ATCC 43063	2146480	NC_014246
Schaalia odontolytica ATCC 17982	2393958	NZ_AAYI02000000
Bifidobacterium adolescentis ATCC 15703	2089645	NZ_BAAD01000000
Bifidobacterium angulatum DSM 20098 = JCM 7096	2008208	NZ_ABYS02000000
Bifidobacterium animalis subsp. lactis AD011	1933695	NC_011835
Bifidobacterium bifidum NCIMB 41171	2202695	NZ_ABQP01000000
Bifidobacterium breve DSM 20213 = JCM 1192	2331386	NZ_ACCG02000000
Bifidobacterium catenulatum DSM 16992 = JCM 1194 = LMG 11043	2058429	NZ_ABXY01000000
Bifidobacterium dentium ATCC 27678	2642081	NZ_ABIX02000000
Bifidobacterium gallicum DSM 20093 = LMG 11596	2004594	NZ_JGYW01000000
Bifidobacterium longum DJO10A	2389526	NC_004253
Bifidobacterium pseudocatenulatum DSM 20438 = JCM 1200 = LMG 10505	2304808	NZ_ABXX02000000
Gardnerella vaginalis 409-05	1617545	NC_013721
Actinospica robiniae DSM 44927	9918887	NZ_AZAN01000000
Catenulispora acidiphila DSM 44928	10467782	NZ_ABTI01000000
Lancefieldella parvulum DSM 20469	1543805	NZ_ABTD01000000
Lancefieldella rimae ATCC 49626	1626291	NZ_ACFE01000000

Collinsella intestinalis DSM 13280	1809497	NZ_ABXH02000000
Collinsella stercoris DSM 13279	2475429	NZ_ABXJ01000000
Coriobacterium glomerans PW2	2115681	NC_015389
Cryptobacterium curtum DSM 15641	1617804	NZ_ABTM01000000
Eggerthella lenta DSM 2243	3632260	NZ_ABTT01000000
Slackia heliotrinireducens DSM 20476	3165038	NZ_ABUP01000000
Corynebacterium accolens ATCC 49725	2437186	NZ_ACGD01000000
Corynebacterium amycolatum SK46	2513912	NZ_ABZU01000000
Corynebacterium aurimucosum ATCC 700975	2819226	NC_010813
Corynebacterium diphtheriae NCTC 13129	2488635	NC_002935
Corynebacterium efficiens YS-314	3219505	NC_004320
Corynebacterium glucuronolyticum ATCC 51867	2809149	NZ_ABYP01000000
Corynebacterium glutamicum ATCC 13032	3309401	NC_003450
Corynebacterium glutamicum R	3363299	NC_009343
Corynebacterium jeikeium K411	2476822	NC_003080
Corynebacterium kroppenstedtii DSM 44385	2446804	NC_012704
Corynebacterium matruchotii ATCC 33806	2992255	NZ_ACEB01000000
Corynebacterium pseudogenitalium ATCC 33035	2601426	NZ_ABYQ02000000
Corynebacterium striatum ATCC 6940	2828991	NZ_ACGE01000000
Corynebacterium urealyticum DSM 7109	2369219	NC_010545
Gordonia bronchialis DSM 43247	5290012	NZ_ABTV01000000
Mycobacterium avium 104	5475491	NC_008595
Mycobacterium tuberculosis H37Rv	4411532	NC_000962
Mycobacterium tuberculosis variant bovis BCG str. Pasteur 1173P2	4374522	NC_008769
Mycobacterium sp. JLS	6048425	AAQC01000000
Mycobacterium sp. KMS	6256079	AAQD01000000
Mycobacterium sp. MCS	5920523	AAPW01000000
Mycobacterium kansasii ATCC 12478	6577228	NZ_CM000636
Mycobacterium leprae Br4923	3268071	FM211192
Mycobacterium marinum M	6660144	NC_010604
Mycobacterium ulcerans Agy99	5805761	NC_005916
Mycobacteroides abscessus ATCC 19977	5090491	NC_010394
Mycolicibacterium gilvum PYR-GCK	5982829	AAPA01000000
Mycolicibacterium smegmatis MC2 155	6988209	NC_008596
Mycolicibacterium vanbaalenii PYR-1	6491865	NZ_AAPF01000000
Nocardia farcinica IFM 10152	6292344	NC_006363
Rhodococcus erythropolis PR4	6895538	NC_007487
Rhodococcus jostii RHA1	9702737	NC_008271
Rhodococcus opacus B4	8834939	NC_012522
Frankia sp. EAN1pec	8982042	AAII01000000
Frankia alni ACN14a	7497934	NC_008278

Frankia casuarinae	5433628	NZ_AAIE01000000
Geodermatophilus obscurus DSM 43160	5322497	NZ_ABTU01000000
Stackebrandtia nassauensis DSM 44728	6841557	NZ_ABUS01000000
Kineococcus radiotolerans SRS30216 = ATCC BAA-149	4956672	NZ_AAEF02000000
Beutenbergia cavernae DSM 12333	4669183	NZ_ABTE01000000
Brevibacterium aurantiacum	4366969	NZ_AAGP01000000
Brachybacterium faecium DSM 4810	3614992	NZ_ABTF01000000
Dermacoccus sp. SAI-028	3141763	NZ_SMFS01000000
Intrasporangium calvum	4025044	NZ_CP031145
Janibacter sp. HTCC2649	4235068	NZ_AAMN01000000
Jonesia denitrificans DSM 20603	2749646	NZ_ABUA01000000
Kytococcus sedentarius DSM 20547	2785024	NZ_ABUD01000000
Clavibacter michiganensis subsp. michiganensis NCPPB 382	3395237	NC_009479
Clavibacter michiganensis subsp. sepedonicus	3403786	NC_010408
Leifsonia xyli subsp. xyli str. CTCB07	2584158	NC_006087
Microbacterium sp. SAI-030	4180068	NZ_VMRZ01000000
Arthrobacter sp. FB24	5070478	NZ_AAHG01000000
Kocuria rhizophila DC2201	2697540	NC_010617
Paenarthrobacter aurescens TC1	5226648	NC_008713
Pseudarthrobacter chlorophenolicus A6	4980870	NZ_ABKU01000000
Renibacterium salmoninarum ATCC 33209	3155250	NC_010168
Rothia mucilaginosa DY-18	2264603	NC_013715
Xylanimonas cellulosilytica DSM 15894	3831380	NZ_ABVC01000000
Sanguibacter keddieii DSM 10542	4253413	NZ_ABUN01000000
Micromonospora sp. ATCC 39149	6819904	NZ_ACES01000000
Salinispora arenicola CNS-205	5786361	AAWA01000000
Salinispora tropica CNB-440	5183331	NZ_AATJ01000000
Nakamurella multipartita DSM 44233	6060298	NZ_ABUH01000000
Kribbella flavida DSM 17836	7579488	NZ_ABUC01000000
Nocardioides sp. JS614	5293685	NZ_AAJB01000000
Cutibacterium acnes KPA171202	2560265	NC_006085
Cutibacterium acnes SK137	2495334	NZ_ABZW01000000
Actinosynnema mirum DSM 43827	8248144	NZ_ABTA01000000
Saccharomonospora viridis DSM 43017	4308349	NZ_ABUM01000000
Saccharopolyspora erythraea NRRL 2338	8212805	NC_009142
Rubrobacter radiotolerans DSM 5868	3398074	NZ_FWWX01000000
Rubrobacter xylanophilus DSM 9941	3225748	NZ_AAEB02000000
Streptomyces coelicolor A3(2)	9054847	AL645771
Streptomyces himastatinicus ATCC 53653	11030030	NZ_ACEX01000000
Streptomyces avermitilis MA-4680 = NBRC 14893	10426843	NZ_BAVY01000000
Streptomyces clavuligerus	6941735	NZ_ABJH01000000

Streptomyces filamentosus NRRL 11379	7851943	NZ_ABYX02000000
Streptomyces griseoflavus Tu4000	8047042	NZ_ACFA01000000
Streptomyces griseus subsp. griseus NBRC 13350	8545929	NC_010572
Streptomyces lividans TK24	8318010	NZ_ACEY01000000
Streptomyces pristinaespiralis ATCC 25486	8133379	NZ_ABJI02000000
Streptomyces scabiei 87.22	10148695	NC_013929
Streptomyces sp. AA4	9175669	ACEV01000000
Streptomyces sp. C	8461333	NZ_ACEW01000000
Streptomyces sp. Mg1	7260368	NZ_ABJF01000000
Streptomyces sp. SPB074	6970553	NZ_ABJG02000000
Streptomyces sp. SPB78	7557384	NZ_ACEU01000000
Streptomyces sviceus ATCC 29083	9313494	NZ_ABJJ02000000
Streptomyces viridochromogenes DSM 40736	8653262	NZ_ACEZ01000000
Streptomyces viridosporus ATCC 14672	8511919	NZ_ABYA01000000
Thermobifida fusca YX	3642249	AAAQ02000000
Streptosporangium roseum DSM 43021	10369518	NZ_ABUU01000000
Thermomonospora curvata DSM 43183	5639016	NZ_ABUZ01000000
Conexibacter woesei DSM 14684	6359369	NZ_ABTL01000000
Aquifex aeolicus VF5	1590791	NC_001880
Hydrogenobacter thermophilus TK-6	1743135	NC_013799
Hydrogenobaculum sp. Y04AAS1	1559514	ABFI01000000
Thermocrinis albus DSM 14484	1500577	NC_013894
Persephonella marina EX-H1	1983966	NC_012439
Sulfurihydrogenibium sp. YO3AOP1	1838442	ABLS01000000
Sulfurihydrogenibium azorense Az-Fu1	1640877	NC_012438
Desulfurobacterium thermolithotrophum DSM 11699	1541968	NC_015185
Caldithrix abyssi DSM 13497	4955439	NZ_AGCQ01000000
Candidatus Cloacimonas acidaminovorans str. Evry	2246820	NC_020449
Candidatus Korarchaeum cryptofilum OPF8	1590757	NC_010482
Aciduliprofundum boonei T469	2981805	ABSD01000000
Ferroplasma acidarmanus Fer1	1935211	NZ_CM000428
Picrophilus torridus DSM 9790	1545895	NC_005877
Thermoplasma acidophilum DSM 1728	1564906	NC_002578
Thermoplasma volcanium GSS1	1584804	NC_002689
Candidatus Protochlamydia amoebophila UWE25	2438912	NC_005861
Chlorobaculum parvum NCIB 8327	2289249	NC_011027
Chlorobium chlorochromatii CaD3	2572079	CP000108
Chlorobium ferrooxidans DSM 13031	2538957	NZ_AASE01000000
Chlorobium limicola DSM 245	2763181	NZ_AAHJ01000000
Chlorobium phaeobacteroides BS1	2736403	AAIC01000000
Chlorobium phaeovibrioides DSM 265	1966858	AAJD01000000

Pelodictyon luteolum DSM 273	2364842	NC_007512
Pelodictyon phaeoclathratiforme BU-1	3018238	NZ_AAIK01000000
Prosthecochloris aestuarii DSM 271	2579695	NZ_AAIJ01000000
Chloroherpeton thalassium ATCC 35110	3293456	NC_011026
Chloroflexus aggregans DSM 9485	4684931	NZ_AAUI01000000
Chloroflexus aurantiacus J-10-fl	5258541	NZ_AAAH02000000
Roseiflexus castenholzii DSM 13941	5723298	NZ_AAUM01000000
Roseiflexus sp. RS-1	5801598	NZ_AAQU01000000
Herpetosiphon aurantiacus DSM 785	6785430	AATI01000000
Dehalococcoides mccartyi 195	1469720	NC_002936
Ktedonobacter racemifer DSM 44963	13661586	NZ_ADVG01000000
Sphaerobacter thermophilus DSM 20745	3993764	NZ_ABUQ01000000
Thermobaculum terrenum ATCC BAA-798	3101581	NZ_ABUX01000000
Thermomicrobium roseum DSM 5159	2920744	NC_011961
Coprothermobacter proteolyticus DSM 5265	1424912	NC_011295
Aeropyrum pernix K1	1669696	NC_000854
Desulfurococcus amylolyticus 1221n	1365223	NC_011766
Desulfurococcus mucosus DSM 2162	1314639	NC_014961
Ignicoccus hospitalis KIN4/I	1297538	NC_009776
Staphylothermus marinus F1	1570485	NC_009033
Hyperthermus butylicus DSM 5456	1667163	NC_008818
Pyrolobus fumarii 1A	1843267	NC_015931
Metallosphaera sedula DSM 5348	2191517	AAVI01000000
Saccharolobus solfataricus P2	2992245	NC_002754
Sulfolobus acidocaldarius DSM 639	2225959	NC_007181
Sulfolobus islandicus L.D.8.5	2748647	NC_013770
Sulfurisphaera tokodaii str. 7	2694756	NC_003106
Thermofilum pendens Hrk 5	1813393	NZ_AASJ01000000
Caldivirga maquilingensis IC-167	2077567	NZ_AAXQ01000000
Pyrobaculum aerophilum str. IM2	2222430	NC_003364
Pyrobaculum arsenaticum DSM 13514	2121076	NC_009376
Pyrobaculum calidifontis JCM 11548	2009313	NC_009073
Pyrobaculum islandicum DSM 4184	1826402	NC_008701
Pyrobaculum neutrophilum V24Sta	1769823	NZ_ABIO01000000
Vulcanisaeta distributa DSM 14429	2374137	NC_014537
Gloeobacter violaceus PCC 7421	4659019	NC_005125
Nodularia spumigena CCY9414	5316258	NZ_AAVW01000000
Nostoc punctiforme PCC 73102	9059191	NZ_AAAY02000000
Nostoc sp. PCC 7120 = FACHB-418	7211789	NC_003241
Trichormus variabilis ATCC 29413	7105752	NZ_AAEA01000000
Crocosphaera chwakensis CCY0110	5880532	NZ_AAXW01000000

Crocosphaera subtropica ATCC 51142	5460377	NC_010542
Gloeothece citriformis PCC 7424	6554169	NZ_ABOY01000000
Gloeothece verrucosa PCC 7822	7841948	NZ_ABVE01000000
Rippkaea orientalis PCC 8801	4787694	NZ_ABLR01000000
Rippkaea orientalis PCC 8802	4803347	ABVI01000000
Microcystis aeruginosa NIES-843	5842795	NC_010296
Cyanothece sp. PCC 7425	5786110	ABVJ01000000
Trichodesmium erythraeum IMS101	7750108	AABK04000000
Lyngbya sp. PCC 8106	7037511	NZ_AAVU01000000
Limnospira maxima CS-328	6003314	NZ_ABYK01000000
Thermosynechococcus vestitus BP-1	2593857	NC_004113
Acaryochloris marina MBIC11017	8361599	NC_009934
Synechocystis sp. PCC 6803	3947019	NC_005232
Parasynechococcus marenigrum WH 8102	2434428	AABA02000000
Prochlorococcus marinus str. AS9601	1669886	NC_008816
Synechococcus elongatus PCC 7942	2742269	NZ_AADZ01000000
Synechococcus sp. BL107	2285034	NZ_AATZ01000000
Synechococcus sp. CC9311	2606748	NC_008319
Synechococcus sp. CC9605	2510659	NC_007516
Synechococcus sp. CC9902	2234828	NC_007513
Synechococcus sp. JA-2-3B'a(2-13)	3046682	NC_007776
Synechococcus sp. JA-3-3Ab	2932766	NC_007775
Synechococcus sp. PCC 7002	3409935	NC_010474
Synechococcus sp. PCC 7335	5973558	NZ_ABRV01000000
Synechococcus sp. RCC307	2224914	CT978603
Synechococcus sp. RS9916	2664873	NZ_AAUA01000000
Synechococcus sp. RS9917	2584918	NZ_AANP01000000
Synechococcus sp. WH 5701	3280236	NZ_AANO01000000
Synechococcus sp. WH 7803	2366980	CT971583
Synechococcus sp. WH 7805	2627046	NZ_AAOK01000000
Calditerrivibrio nitroreducens DSM 19672	2216552	NC_014749
Deferribacter desulfuricans SSM1	2542933	NC_013940
Flexistipes sinusarabici DSM 4947	2526590	NC_015672
Denitrovibrio acetiphilus DSM 12809	3222077	NZ_ABTN01000000
Deinococcus deserti VCD115	3855329	NC_012528
Deinococcus geothermalis DSM 11300	3247018	NZ_AAHE01000000
Deinococcus hopiensis KR-140	6650868	NZ_FWWU01000000
Deinococcus maricopensis DSM 21211	3498530	NC_014958
Deinococcus proteolyticus MRP	2886836	NC_015163
Deinococcus radiodurans R1	3284156	NC_000958
Marinithermus hydrothermalis DSM 14884	2269167	NC_015387

Meiothermus ruber DSM 1279	3097457	NZ_ABUF01000000
Oceanithermus profundus DSM 14977	2439291	NC_014753
Thermus aquaticus Y51MC23	2338641	NZ_CP010826
Thermus thermophilus HB27	2127482	NC_005838
Dictyoglomus thermophilum H-6-12	1959987	NC_011297
Dictyoglomus turgidum DSM 6724	1855560	NZ_ABYR01000000
Elusimicrobium minutum Pei191	1643562	NZ_ABIQ01000000
Archaeoglobus fulgidus DSM 4304	2178400	NC_000917
Archaeoglobus profundus DSM 5631	1563423	NC_013742
Archaeoglobus veneficus SNP6	1901943	NC_015320
Ferroglobus placidus DSM 10642	2196266	NZ_ACYZ01000000
Methanobrevibacter ruminantium M1	2937203	NC_013790
Methanobrevibacter smithii ATCC 35061	1853160	NC_009515
Methanosphaera stadtmanae DSM 3091	1767403	NC_007681
Methanothermobacter thermautotrophicus str. Delta H	1751377	NC_000916
Methanothermus fervidus DSM 2088	1243342	NC_014658
Methanocaldococcus fervens AG86	1507251	NZ_ACQW01000000
Methanocaldococcus jannaschii DSM 2661	1739927	NC_001733
Methanocaldococcus sp. FS406-22	1773136	NZ_ADCA01000000
Methanocaldococcus vulcanius M7	1761737	NZ_ACUW01000000
Methanococcus aeolicus Nankai-3	1569500	NZ_AAZL01000000
Methanococcus maripaludis	1714918	NZ_CP026606
Methanococcus vannielii SB	1720048	NZ_AAWX01000000
Methanococcus voltae A3	1936387	ABHB01000000
Methanopyrus kandleri AV19	1694969	NC_003551
Haloarcula marismortui ATCC 43049	4274642	NC_006395
Halomicrobium mukohataei DSM 12286	3332349	NZ_ABTY01000000
Halorhabdus utahensis DSM 12940	3116795	NZ_ABTZ01000000
Natronomonas pharaonis DSM 2160	2749696	NC_007427
Halobacterium salinarum NRC-1	2571010	NC_002607
Haloferax volcanii DS2	4012900	NC_013966
Halogeometricum borinquense DSM 11551	3944467	NZ_ABTX01000000
Haloquadratum walsbyi DSM 16790	3179361	NC_008213
Halorubrum lacusprofundi ATCC 49239	3692576	NZ_ABEB01000000
Haloterrigena turkmenica DSM 5511	5440782	NC_013749
Natrialba magadii ATCC 43099	4443643	NZ_ACIT01000000
Natronococcus occultus SP4	4314118	NC_019976
Methanocella paludicola SANAE	2957635	NC_013665
Methanocorpusculum labreanum Z	1804962	NC_008942
Methanoculleus marisnigri JR1	2478101	NZ_AASI01000000
Methanolacinia petrolearia DSM 11571	2843290	NC_014507

Methanoregula boonei 6A8	2542943	NC_009712
Methanosphaerula palustris E1-9c	2922917	NZ_ABZB01000000
Methanospirillum hungatei JF-1	3544738	NZ_AALU01000000
Methanococcoides burtonii DSM 6242	2575032	NZ_AADH02000000
Methanohalophilus mahii DSM 5219	2012424	NC_014002
Methanomethylovorans hollandica DSM 15978	2714013	NC_019972
Methanosalsum zhilinae DSM 4017	2138444	NC_015676
Methanosarcina acetivorans C2A	5751492	AE010299
Methanosarcina barkeri str. Fusaro	4873766	AAAR03000000
Methanosarcina mazei Go1	4096345	NC_003901
Methanothrix thermoacetophila PT	1879471	NZ_AAOR01000000
Palaeococcus ferrophilus DSM 13482	2206431	NZ_LANF01000000
Pyrococcus abyssi GE5	1768562	NC_001773
Pyrococcus furiosus DSM 3638	1908256	NC_003413
Pyrococcus horikoshii OT3	1738505	NC_000961
Thermococcus gammatolerans EJ3	2045438	NC_012804
Thermococcus kodakarensis KOD1	2088737	NC_006624
Thermococcus onnurineus NA1	1847607	NC_011529
Thermococcus sibiricus MM 739	1845800	NC_012883
Fibrobacter succinogenes subsp. succinogenes S85	3842635	NZ_ACRA01000000
Alicyclobacillus acidocaldarius subsp. acidocaldarius DSM 446	3205686	NZ_ABTB01000000
Kyrpidia tusciae DSM 2912	3384766	NC_014098
Alkalihalobacillus clausii KSM-K16	4303871	NC_006582
Alkalihalophilus pseudofirmus OF4	4249248	NC_013793
Anoxybacillus flavithermus WK1	2846746	NC_011567
[Bacillus thuringiensis] serovar konkukian str. 97-27	5314794	NC_006578
Bacillus anthracis str. A0248	5503926	NC_012655
Bacillus cereus B4264	5419036	NZ_ABDI01000000
Bacillus coahuilensis m4-4	3381351	NZ_ABFU01000000
Bacillus cytotoxicus NVH 391-98	4094159	NZ_AALL01000000
Bacillus licheniformis DSM 13 = ATCC 14580	4222645	NC_006322
Bacillus mycoides KBAB4	5872743	NZ_AAOY01000000
Bacillus pumilus SAFR-032	3704641	NC_009848
Bacillus sp. B14905	4497271	AAXV01000000
Bacillus sp. NRRL B-14911	5116500	NZ_AAOX01000000
Bacillus subtilis subsp. subtilis str. 168	4215606	NC_000964
Bacillus thuringiensis str. Al Hakam	5313030	NC_008598
Bacillus velezensis FZB42	3918596	NC_009725
Geobacillus kaustophilus HTA426	3592666	NC_006509
Geobacillus sp. WCH70	3508804	ABLJ01000000
Geobacillus sp. Y412MC61	3667901	ACED01000000

Geobacillus thermodenitrificans NG80-2	3608012	NC_009329
Halalkalibacterium halodurans C-125	4202352	NC_002570
Lysinibacillus sphaericus C3-41	4817463	NC_010381
Oceanobacillus iheyensis HTE831	3630528	NC_004193
Priestia megaterium DSM 319	5097447	NC_014103
[Bacillus] selenitireducens MLS10	3592487	NZ_ABHZ01000000
Weizmannia coagulans 36D1	3552226	NZ_AAWV02000000
Gemella haemolysans ATCC 10379	1916192	NZ_ACDZ02000000
Exiguobacterium sibiricum 255-15	3040786	NZ_AADW02000000
Exiguobacterium sp. AT1b	2999895	NZ_ABPF01000000
Listeria grayi DSM 20601	2598321	NZ_ACCR02000000
Listeria innocua Clip11262	3093113	NC_003383
Listeria monocytogenes EGD-e	2944528	NC_003210
Listeria seeligeri serovar 1/2b str. SLCC3954	2797636	NC_013891
Listeria welshimeri serovar 6b str. SLCC5334	2814130	NC_008555
Brevibacillus brevis NBRC 100599	6296436	NC_012491
Paenibacillus sp. JDR-2	7184930	ABKS01000000
Paenibacillus sp. Y412MC10	7121665	NZ_ABRG01000000
Macrococcus caseolyticus JCSC5402	2219737	NC_012003
Staphylococcus aureus subsp. aureus Mu50	2903636	NC_002774
Staphylococcus capitis SK14	2435835	NZ_ACFR01000000
Staphylococcus carnosus subsp. carnosus TM300	2566424	NC_012121
Staphylococcus epidermidis ATCC 12228	2564615	NC_005003
Staphylococcus haemolyticus JCSC1435	2697861	NC_007171
Staphylococcus lugdunensis HKU09-01	2658366	NC_013893
Staphylococcus saprophyticus subsp. saprophyticus ATCC 15305 = NCTC 7292	2577899	NC_007352
Carnobacterium sp. AT7	2442118	NZ_ABHH01000000
Enterococcus faecalis OG1RF	2739625	NZ_ABPI01000000
Enterococcus faecium DO	2848380	NZ_AAAK03000000
Lacticaseibacillus paracasei	3079196	NC_010999
Lacticaseibacillus rhamnosus GG	3010111	NC_013198
Lactiplantibacillus plantarum JDM1	3197759	NC_012984
Lactobacillus acidophilus NCFM	1993560	NC_006814
Lactobacillus crispatus ST1	2043161	NC_014106
Lactobacillus delbrueckii subsp. bulgaricus ATCC 11842 = JCM 1002	1864998	NC_008054
Lactobacillus gasseri ATCC 33323 = JCM 1131	1894360	NZ_AAAO02000000
Lactobacillus helveticus DPC 4571	2080931	NC_010080
Lactobacillus jensenii 1153	1746219	ABWG02000000
Lactobacillus johnsonii NCC 533	1992676	NC_005362
Lactobacillus paragasseri JV-V03	2011855	NZ_ACGO02000000

Lactobacillus ultunensis DSM 16047	2248406	NZ_ACGU01000000
Latilactobacillus sakei subsp. sakei 23K	1884661	NC_007576
Lentilactobacillus buchneri ATCC 11577	2906028	ACGH01000000
Lentilactobacillus hilgardii DSM 20176 = ATCC 8290	2721039	NZ_ACGP01000000
Leuconostoc citreum KM20	1896614	NC_010469
Leuconostoc mesenteroides subsp. mesenteroides ATCC 8293	2075763	NZ_AABH02000000
Levilactobacillus brevis ATCC 367	2340228	NC_008499
Ligilactobacillus ruminis ATCC 25644	2108740	NZ_ACGS02000000
Ligilactobacillus salivarius UCC118	2133977	NC_006530
Limosilactobacillus fermentum IFO 3956	2098685	NC_010610
Limosilactobacillus reuteri JCM 1112	2039414	NC_010609
Limosilactobacillus vaginalis DSM 5837 = ATCC 49540	1877332	NZ_ACGV01000000
Oenococcus oeni PSU-1	1780517	NZ_AABJ03000000
Pediococcus pentosaceus ATCC 25745	1832387	NZ_AAEV01000000
Lactococcus lactis subsp. cremoris MG1363	2529478	NC_009004
Streptococcus agalactiae 515	2032743	NZ_CP051004
Streptococcus dysgalactiae subsp. equisimilis GGS_124	2106340	NC_012891
Streptococcus equi subsp. equi 4047	2253793	NC_012471
Streptococcus gallolyticus UCN34	2350911	NC_013798
Streptococcus gordonii str. Challis substr. CH1	2196662	NC_009785
Streptococcus infantarius subsp. infantarius ATCC BAA-102	1925187	NZ_ABJK02000000
Streptococcus mitis B6	2146611	NC_013853
Streptococcus mutans UA159	2032925	NC_004350
Streptococcus pneumoniae ATCC 700669	2221315	NC_011900
Streptococcus pyogenes M1 GAS	1852433	NC_002737
Streptococcus sanguinis SK36	2388435	NC_009009
Streptococcus suis 05ZYH33	2096309	CP000407
Streptococcus thermophilus CNRZ1066	1796226	NC_006449
Streptococcus uberis 0140J	1852352	NC_012004
Clostridium sp. L2-50	2954616	NZ_AAYW02000000
[Clostridium] leptum DSM 753	3196486	NOXF01000000
Alkaliphilus metalliredigens QYMF	4929566	NZ_AAKU01000000
Alkaliphilus oremlandii OhILAs	3123558	NZ_AAQV01000000
Clostridium acetobutylicum ATCC 824	4132880	NC_001988
Clostridium beijerinckii NCIMB 8052	6000632	NZ_AALO01000000
Clostridium botulinum A str. ATCC 19397	3863450	NC_009697
Clostridium butyricum 5521	4540699	NZ_ABDT01000000
Clostridium kluyveri DSM 555	4023800	NC_009466
Clostridium novyi NT	2547720	NC_008593
Clostridium perfringens ATCC 13124	3256683	NC_008261
Clostridium sp. 7_2_43FAA	3813122	NZ_ACDK02000000

Clostridium sp. SS2/1	3142181	ABGC03000000
Clostridium sporogenes ATCC 15579	4102325	NZ_ABKW02000000
Clostridium tetani E88	2873333	NC_004565
Sulfobacillus acidophilus DSM 10332	3557831	CP003180
Thermaerobacter marianensis DSM 12885	2844696	NC_014831
Thermaerobacter subterraneus DSM 13965	2888741	NZ_AENY02000000
Desulfitobacterium hafniense DCB-2	5279134	NZ_AAAW04000000
Syntrophobotulus glycolicus DSM 8271	3406739	NC_015172
Pelotomaculum thermopropionicum SI	3025375	BAAC01000000
Anaerofustis stercorihominis DSM 17244	2284703	NZ_ABIL02000000
Heliomicrobium modesticaldum Ice1	3075407	NC_010337
Anaerobutyricum hallii DSM 3353	3290996	NZ_ACEP01000000
Anaerostipes caccae L1-92	3606936	NZ_ABAX03000000
Blautia hansenii DSM 20583	3058721	NZ_ABYU02000000
Blautia hydrogenotrophica DSM 10507	3626943	NZ_ACBZ01000000
Coprococcus eutactus ATCC 27759	3102987	NZ_ABEY02000000
Dorea formicigenerans ATCC 27755	3186031	NZ_AAXA02000000
Dorea longicatena DSM 13814	2915433	NZ_AAXB02000000
[Clostridium] asparagiforme DSM 15981	6417332	NZ_ACCJ01000000
Enterocloster bolteae ATCC BAA-613	6557988	NZ_ABCC02000000
Epulopiscium sp. 'N.t. morphotype B'	2686047	NZ_ABEQ01000000
[Eubacterium] eligens ATCC 27750	2831389	NC_012780
[Eubacterium] rectale ATCC 33656	3449685	CP001107
Marvinbryantia formatexigens DSM 14469	4548960	NZ_ACCL02000000
[Ruminococcus] lactaris ATCC 29176	2731235	NZ_ABOU02000000
Roseburia intestinalis L1-82	4411375	NZ_ABYJ02000000
Tyzzerella nexilis DSM 1787	3995628	ABWO01000000
[Clostridium] hylemonae DSM 15053	3889859	NZ_ABYI02000000
[Clostridium] scindens ATCC 35704	3622605	NZ_ABFY02000000
Lachnoclostridium phytofermentans ISDg	4847594	NZ_AAQT01000000
Acetivibrio thermocellus JW20	3767546	ABVG02000000
Anaerotruncus colihominis DSM 17241	3719688	NZ_ABGD02000000
Faecalibacterium prausnitzii M21/2	3127383	NZ_ABED02000000
Mageeibacillus indolicus UPII9-5	1809746	NC_013895
[Clostridium] methylpentosum DSM 5476	3478423	ACEC01000000
Pseudoflavonifractor capillosus ATCC 29799	4241076	NZ_AAXG02000000
Ruminiclostridium cellulolyticum H10	4068724	NZ_AAVC01000000
Subdoligranulum variabile DSM 15176	3245471	NZ_ACBY02000000
Candidatus Desulforudis audaxviator MP104C	2349476	NC_010424
Desulfofarcimen acetoxidans DSM 771	4545624	NZ_ABTQ01000000
Desulfonispora thiosulfatigenes DSM 11270	2412168	NZ_FWWT01000000

Desulforamulus reducens MI-1	3608104	NZ_AAOP01000000
Clostridioides difficile 630	4274782	NZ_CP010905
Intestinibacter bartlettii DSM 16795	2972256	NZ_ABEZ02000000
[Clostridium] hiranonis DSM 13275	2479772	NZ_ABWP01000000
Symbiobacterium thermophilum IAM 14863	3566135	NC_006177
Syntrophomonas wolfei subsp. wolfei str. Goettingen G311	2936195	NZ_AAJG01000000
[Bacteroides] pectinophilus ATCC 43243	3028326	ABVQ01000000
Clostridiales bacterium 1_7_47FAA	6548966	ABQR01000000
Halanaerobium praevalens DSM 2228	2309262	NC_017455
Halothermothrix orenii H 168	2578146	NZ_AAOZ01000000
Acetohalobium arabaticum DSM 5501	2469596	NC_014378
Halobacteroides halobius DSM 5150	2649255	NC_019978
Natranaerobius thermophilus JW/NM-WN-LF	3191453	NZ_ABKR01000000
Caldanaerobacter subterraneus subsp. tengcongensis MB4	2689445	NC_003869
Carboxydothermus hydrogenoformans Z-2901	2401520	NC_007503
Ammonifex degensii KC4	2157067	NC_013386
Moorella thermoacetica ATCC 39073	2628784	AADT03000000
Thermoanaerobacter italicus Ab9	2451061	NZ_ACVH01000000
Thermoanaerobacter pseudethanolicus ATCC 33223	2362816	NZ_AAKQ01000000
Thermoanaerobacter sp. X514	2457259	NZ_AATV01000000
Caldicellulosiruptor bescii DSM 6725	2931662	NZ_ABYZ01000000
Caldicellulosiruptor saccharolyticus DSM 8903	2970275	NZ_AALW01000000
Mahella australiensis 50-1 BON	3135972	NC_015520
Thermodesulfobium narugense DSM 14796	1898865	NC_015499
Coprobacillus sp. D7	3527730	ACDT02000000
Amedibacillus dolichus DSM 3991	2191053	NZ_ABAW02000000
[Clostridium] spiroforme DSM 1552	2507885	NZ_ABIK02000000
Erysipelatoclostridium ramosum DSM 1402	3235195	NZ_ABFX02000000
Holdemanella biformis DSM 3989	2517763	NZ_ABYT01000000
Holdemania filiformis DSM 12042	3932923	NZ_ACCF01000000
Acidaminococcus fermentans DSM 20731	2329769	NC_013740
Mitsuokella multacida DSM 20544	2577056	NZ_ABWK02000000
Selenomonas sputigena ATCC 35185	2559453	NZ_ACKP02000000
Thermosinus carboxydivorans Nor1	2889774	NZ_AAWL01000000
Veillonella parvula DSM 2008	2132142	NZ_ABVB01000000
Anaerococcus hydrogenalis DSM 7454	1889366	NZ_ABXA01000000
Anaerococcus prevotii DSM 20548	1998633	NZ_ABTC01000000
Anaerococcus tetradius ATCC 35098	2145347	NZ_ACGC01000000
Finegoldia magna ATCC 29328	1986740	NC_010371
Parvimonas micra ATCC 33270	1703872	NZ_ABEE02000000
Peptoniphilus asaccharolyticus DSM 20463	2232586	NZ_FWWR01000000

Fusobacterium gonidiaformans ATCC 25563	1698329	NZ_ACET02000000
Fusobacterium mortiferum ATCC 9817	2671036	NZ_ACDB02000000
Fusobacterium necrophorum D12	1960925	NZ_ACDG02000000
Fusobacterium nucleatum subsp. nucleatum ATCC 25586	2174500	AE009951
Fusobacterium periodonticum 2_1_31	2546312	NZ_ACDC03000000
Fusobacterium ulcerans ATCC 49185	3487504	NZ_ACDH02000000
Ilyobacter polytropus DSM 2926	3132314	NC_014634
Leptotrichia buccalis C-1013-b	2465610	NZ_ABUE01000000
Pseudoleptotrichia goodfellowii DSM 19756	2281162	NZ_AZXW01000000
Sebaldella termitidis ATCC 33386	4486650	NZ_ABUO01000000
Streptobacillus moniliformis DSM 12112	1673280	NZ_ABUT01000000
Gemmatimonas aurantiaca T-27	4636964	NC_012489
Lentisphaera araneosa HTCC2155	6023180	NZ_ABCK01000000
Thermodesulfovibrio yellowstonii DSM 11347	2003803	NC_011296
Isosphaera pallida ATCC 43644	5529304	NC_014957
Blastopirellula marina DSM 3645	6663851	NZ_AANZ01000000
Pirellula staleyi DSM 6068	6196199	NC_013720
Rhodopirellula baltica SH 1	7145576	NC_005027
Gimesia maris DSM 8797	7777997	NZ_ABCE01000000
Rubinisphaera brasiliensis DSM 5305	6006602	NZ_AEIC01000000
Acidithiobacillus ferrooxidans ATCC 53993	2885038	NC_011206
Brevundimonas sp. GW460-12-10-14-LB2	3263775	NZ_CP015511
Caulobacter segnis ATCC 21756	4655622	NZ_ADBN01000000
Caulobacter sp. K31	5889399	AATH01000000
Caulobacter vibrioides NA1000	4042929	NC_011916
Phenylobacterium zucineum HLK1	4379231	NC_011143
Aurantimonas manganoxydans SI85-9A1	4326814	NZ_AAPJ01000000
Fulvimarina pelagi HTCC2506	3806238	NZ_AATP01000000
Bartonella bacilliformis KC583	1445021	NZ_AANF01000000
Bartonella grahamii as4aup	2369520	NC_012847
Bartonella henselae str. Houston-1	1931047	NC_005956
Bartonella quintana str. Toulouse	1581384	NC_005955
Bartonella tribocorum CIP 105476	2642404	NC_010160
Beijerinckia indica subsp. indica ATCC 9039	4418616	NC_010578
Methylocella silvestris BL2	4305430	NZ_ABLP01000000
Rhodopseudomonas palustris BisA53	5505494	AALA01000000
Brucella abortus 2308	3278307	NC_007624
Brucella anthropi ATCC 49188	5205777	NC_009672
Brucella canis ATCC 23365	3312769	NC_010104
Brucella ceti B1/94	3337159	NZ_ACEK01000000
Brucella melitensis ATCC 23457	3311219	NC_012442

Brucella microti CCM 4915	3337369	NC_013118
Brucella neotomae 5K33	3329623	NZ_ACEH01000000
Brucella ovis ATCC 25840	3275590	NC_009504
Brucella pinnipedialis M292/94/1	3373519	NZ_ACEF01000000
Brucella sp. 83/13	3153851	NZ_ACBQ01000000
Brucella suis ATCC 23445	3324607	NC_010167
Methylobacterium nodulans ORS 2060	8839022	NZ_ABIP01000000
Methylobacterium radiotolerans JCM 2831	6899110	NC_010507
Methylobacterium sp. 4-46	7737025	ABAY01000000
Methylorubrum extorquens AM1	6879778	NC_012810
Methylorubrum populi BJ001	5848997	NZ_ABFR01000000
Afipia carboxidovorans OM5	3745629	NZ_CM000486
Bradyrhizobium diazoefficiens USDA 110	9105828	NC_004463
Bradyrhizobium sp. BTAi1	8493513	NZ_AALJ01000000
Bradyrhizobium sp. ORS 278	7456587	NC_009445
Nitrobacter hamburgensis X14	5011522	NZ_AAIS01000000
Nitrobacter sp. Nb-311A	4107280	NZ_AAMY01000000
Nitrobacter winogradskyi Nb-255	3402093	NC_007406
Rhodopseudomonas palustris BisB5	4892717	AAKZ01000000
Parvibaculum lavamentivorans DS-1	3914745	NZ_AAWJ01000000
Chelativorans sp. BNC1	4935185	AAED02000000
Hoeflea phototrophica DFL-43	4468465	NZ_ABIA03000000
Mesorhizobium japonicum MAFF 303099	7596297	NC_002682
Candidatus Liberibacter asiaticus str. psy62	1227328	NZ_ABQW01000000
Agrobacterium radiobacter K84	7273300	NC_011987
Agrobacterium vitis S4	6320946	NC_011982
Rhizobium etli 8C-3	7309118	NZ_CP017244
Rhizobium leguminosarum bv. trifolii WSM1325	7418122	NZ_ABJS01000000
Rhizobium phaseoli Brasil 5	6665454	NZ_ABQZ01000000
Rhizobium sp. CIAT894	6657947	NZ_ABRD01000000
Rhizobium sp. Kim5	6817255	NZ_ABQY01000000
Sinorhizobium fredii NGR234	6891900	CP000874
Sinorhizobium medicae WSM419	6817576	NZ_AATG01000000
Pseudovibrio sp. JE062	5726521	NZ_ABXL01000000
Roseibium aggregatum IAM 12614	6561391	NZ_AAUW01000000
Azorhizobium caulinodans ORS 571	5369772	NC_009937
Xanthobacter autotrophicus Py2	5625098	AAPC01000000
Hirschia baltica ATCC 49814	3540114	NC_012983
Hyphomonas neptunium ATCC 15444	3705021	NC_008358
Magnetococcus marinus MC-1	4719581	NZ_AAAN03000000
Maricaulis maris MCS10	3368780	NZ_AASK01000000

Oceanicaulis sp. HTCC2633	3172051	NZ_AAMQ01000000
Parvularcula bermudensis HTCC2503	2902643	NZ_CH724133
Candidatus Pelagibacter ubique HTCC1062	1308759	NC_007205
Brucella pinnipedialis M163/99/10	3404050	NZ_ACBM01000000
Methylobacterium sp. UNC378MF	6358020	NZ_FMWU01000000
Agrobacterium fabrum str. C58	5674258	NC_003062
Sinorhizobium meliloti 1021	6691694	NC_003047
Cereibacter sphaeroides 2.4.1	4602977	NZ_AAAE01000000
Dinoroseobacter shibae DFL 12 = DSM 16493	4417868	NZ_AAVE01000000
Paracoccus denitrificans PD1222	5236194	NZ_AAIT01000000
Phaeobacter inhibens DSM 17395	4227134	NC_018287
Pseudooceanicola batsensis HTCC2597	4440389	NZ_AAMO01000000
Yoonia vestfoldensis SKA53	3066852	NZ_AAMS01000000
Rhodobacteraceae bacterium HTCC2083	4018415	ABXE01000000
Rhodobacteraceae bacterium HTCC2150	3582902	AAXZ01000000
Rhodobacteraceae bacterium KLH11	4487498	ACCW01000000
Jannaschia sp. CCS1	4404049	NZ_AAIG01000000
Maritimibacter alkaliphilus HTCC2654	4536113	NZ_AAMT01000000
Oceanicola granulosus HTCC2516	4053139	NZ_AAOT01000000
Octadecabacter antarcticus 307	4875481	NC_020907
Phaeobacter gallaeciensis DSM 26640	4540155	NC_023138
Phaeobacter inhibens 2.10	4160918	NC_018423
Roseobacter denitrificans OCh 114	4331234	NC_008389
Roseobacter litoralis Och 149	4745450	NC_015741
Roseobacter sp. AzwK-3b	4178704	ABCR01000000
Roseobacter sp. CCS2	3497325	NZ_AAYB01000000
Roseobacter sp. GAI101	4527951	NZ_ABXS01000000
Roseobacter sp. MED193	4674070	NZ_AANB01000000
Roseobacter sp. SK209-2-6	4555826	NZ_AAYC01000000
Roseovarius nubinhibens ISM	3676244	NZ_AALY01000000
Roseovarius sp. 217	4770736	NZ_AAMV01000000
Roseovarius sp. TM1035	4209812	NZ_ABCL01000000
Ruegeria pomeroyi DSS-3	4601048	NC_006569
Ruegeria sp. R11	3821839	ABXM01000000
Ruegeria sp. TM1040	4153699	NZ_AAFG02000000
Sagittula stellata E-37	5262893	NZ_AAYA01000000
Salipiger bermudensis HTCC2601	5482983	NZ_AATQ01000000
Sulfitobacter indolifex HEL-45	4105524	NZ_ABID01000000
Sulfitobacter sp. EE-36	3548498	NZ_AALV01000000
Sulfitobacter sp. NAS-14.1	4010516	NZ_AALZ01000000
Rhodobacterales bacterium HTCC2255	2296803	AATR01000000

Rhodobacterales bacterium Y4I	4344244	ABXF01000000
Acetobacter pasteurianus IFO 3283-01	3340249	NC_013215
Acidiphilium cryptum JF-5	3963080	NZ_AAOO01000000
Gluconacetobacter diazotrophicus PA1 5	3914947	NZ_ABPH01000000
Gluconobacter oxydans 621H	2922384	NC_006676
Granulibacter bethesdensis CGDNIH1	2708434	NC_008343
Azospirillum sp. B510	7599738	NC_013860
Azospirillum brasilense Sp245	7530241	NC_016597
Magnetospirillum magneticum AMB-1	4967148	NC_007626
Magnetospirillum magnetotacticum MS-1	4523935	NZ_JXSL01000000
Rhodospirillum centenum SW	4355543	NC_011420
Rhodospirillum rubrum ATCC 11170	4406557	NZ_AAAG02000000
Erythrobacter litoralis HTCC2594	3052398	NZ_CM000156
Erythrobacter sp. NAP1	3266173	NZ_AAMW01000000
Erythrobacter sp. SD-21	2970874	NZ_ABCG01000000
Novosphingobium aromaticivorans DSM 12444	4233314	NZ_AAAV03000000
Rhizorhabdus wittichii RW1	5915246	AAVK01000000
Sphingobium japonicum UT26S	4424862	NC_014009
Sphingobium sp. GW456-12-10-14-TSB1	5078579	NZ_NGUN01000000
Sphingomonas koreensis	4398689	NZ_PGEN01000000
Sphingomonas sp. SKA58	3955737	NZ_AAQG01000000
Sphingopyxis alaskensis RB2256	3373713	NZ_AAIP01000000
Sphingopyxis sp. GW247-27LB	4791544	NZ_NIWD01000000
Zymomonas mobilis subsp. mobilis ZM4 = ATCC 31821	2056363	NC_006526
Bordetella avium 197N	3732255	NC_010645
Bordetella bronchiseptica RB50	5339179	NC_002927
Bordetella parapertussis 12822	4773551	NC_002928
Bordetella pertussis Tohama I	4086189	NC_002929
Bordetella petrii	5287950	NC_010170
Burkholderia ambifaria AMMD	7528567	NZ_AAJL01000000
Burkholderia cenocepacia AU 1054	7279116	AAHI01000000
Burkholderia dolosa AU0158	6420400	NZ_AAKY01000000
Burkholderia glumae BGR1	7284636	NC_012725
Burkholderia lata	8676277	NZ_AAEI01000000
Burkholderia mallei ATCC 23344	5835527	NC_006349
Burkholderia multivorans ATCC 17616	7008810	NC_010802
Burkholderia orbicola MC0-3	7971389	NZ_AAVA01000000
Burkholderia pseudomallei 1106a	7089249	NZ_AAMA02000000
Burkholderia thailandensis E264	6723972	NC_007650
Burkholderia vietnamiensis G4	8391070	AAEH02000000
Cupriavidus basilensis	8421483	NZ_CP010537

Cupriavidus metallidurans CH34	6913352	NC_007971
Cupriavidus necator H16	7416678	NC_005241
Cupriavidus pinatubonensis JMP134	7255290	AADY01000000
Cupriavidus taiwanensis LMG 19424	6476522	NC_010529
Limnobacter sp. MED105	3390271	NZ_ABCT01000000
Paraburkholderia atlantica	7884858	NZ_ADCC01000000
Paraburkholderia caledonica	7381819	NZ_ARSZ01000000
Paraburkholderia graminis C4D1M	7477263	NZ_ABLD01000000
Paraburkholderia phymatum STM815	8676562	NZ_AAUG01000000
Paraburkholderia phytofirmans PsJN	8214658	NZ_AAUH01000000
Paraburkholderia xenovorans LB400	9731138	NZ_AAAJ03000000
Polynucleobacter asymbioticus QLW-P1DMWA-1	2159490	NZ_AAUC01000000
Polynucleobacter necessarius STIR1	1560469	ABJA01000000
Ralstonia pickettii 12D	5685358	ABDZ01000000
Ralstonia solanacearum GMI1000	5810922	NC_003295
Ralstonia sp. UNC404CL21Col	5087452	NZ_JIBE01000000
Acidovorax citrulli AAC00-1	5352772	NZ_AASX01000000
Acidovorax sp. GW101-3H11	5442520	NZ_LUKZ01000000
Acidovorax sp. JS42	4585154	AASD01000000
Comamonas testosteroni KF-1	6026527	NZ_AAUJ02000000
Comamonas thiooxydans	5373644	NC_013446
Delftia acidovorans SPH-1	6767514	NZ_AAVD01000000
Delftia sp. GW456-R20	6659907	NZ_LWCN01000000
[Acidovorax] ebreus TPSY	3796573	ACCM01000000
Polaromonas naphthalenivorans CJ2	5366143	NZ_AANM01000000
Polaromonas sp. JS666	5898676	NZ_AAFQ02000000
Rhodoferax ferrireducens T118	4969784	NZ_AAJK01000000
Variovorax paradoxus S110	6754997	ACEE01000000
Verminephrobacter eiseniae EF01-2	5597943	NZ_AASQ01000000
Leptothrix cholodnii SP-6	4909403	NC_010524
Herbaspirillum seropedicae SmR1	5513887	NC_014323
Herminiimonas arsenicoxydans	3424307	CU207211
Janthinobacterium sp. Marseille	4110251	NC_009659
Oxalobacter formigenes HOxBLS	2488386	NZ_ACDP02000000
Methylibium petroleiphilum PM1	4643639	NZ_AAEM01000000
Chromobacterium violaceum ATCC 12472	4751080	NC_005085
Laribacter hongkongensis HLHK9	3169329	NC_012559
Eikenella corrodens ATCC 23834	2171245	NZ_ACEA01000000
Neisseria cinerea ATCC 14685	1872773	NZ_ACDY02000000
Neisseria flavescens NRL30031/H210	2205588	NZ_ACEN01000000
Neisseria gonorrhoeae FA 1090	2153922	NC_002946

Neisseria lactamica ATCC 23970	2171992	NZ_ACEQ02000000
Neisseria meningitidis MC58	2272360	NC_003112
Neisseria mucosa ATCC 25996	2578748	NZ_ACDX02000000
Neisseria subflava NJ9703	2292986	ACEO02000000
Sideroxydans lithotrophicus ES-1	3003656	NZ_ACVF01000000
Methylobacillus flagellatus KT	2971517	NZ_AADX02000000
Methylotenera mobilis JLW8	2547570	NC_012968
Methylovorus glucosetrophus SIP3-4	3082007	NC_012972
Nitrosomonas europaea ATCC 19718	2812094	BX321856
Nitrosospira multiformis ATCC 25196	3234309	NC_007617
Methylophilales bacterium HTCC2181	1304428	AAUX01000000
Thiobacillus denitrificans ATCC 25259	2909809	NZ_AAFH01000000
Dechloromonas aromatica RCB	4501104	AADF01000000
Aromatoleum aromaticum EbN1	4727255	NC_006824
Azospira oryzae PS	3806980	NZ_ADDK02000000
Azoarcus olearius	4376040	NC_008702
Thauera sp. MZ1T	4574586	NZ_ABKT01000000
Desulfatibacillum aliphaticivorans	6517073	NZ_ABII01000000
Desulforapulum autotrophicum HRM2	5657782	NC_012109
Desulfobulbus propionicus DSM 2032	3851869	NC_014972
Desulfotalea psychrophila LSv54	3659634	NC_006140
Desulfococcus oleovorans Hxd3	3944167	NZ_AAWN01000000
Desulfohalobium retbaense DSM 5692	2909567	NZ_ABTO01000000
Desulfomicrobium baculatum DSM 4028	3942657	NZ_ABTP01000000
Desulfovibrio alaskensis G20	3730232	NZ_AABN02000000
Desulfovibrio desulfuricans ATCC 27774	2873437	ACCT01000000
Desulfovibrio piger ATCC 29098	2867216	NZ_ABXU01000000
Desulfovibrio vulgaris str. Hildenborough	3773159	NC_005863
Lawsonia intracellularis PHE/MN1-00	1719014	NC_008014
Maridesulfovibrio salexigens DSM 2638	4289847	NZ_ACCN01000000
Solidesulfovibrio magneticus RS-1	5315620	NC_012795
Hippea maritima DSM 10411	1694430	NC_015318
Desulfuromonas acetoxidans DSM 684	3828328	NZ_AAEW02000000
Pelobacter propionicus DSM 2379	4241119	NZ_AAJH01000000
Citrifermentans bemidjiense Bem	4615150	NZ_AAZA01000000
Geobacter metallireducens GS-15	4011182	NC_007515
Geobacter sp. M21	4745806	ABRF01000000
Geobacter sulfurreducens PCA	3814128	NC_002939
Geotalea daltonii FRC-32	4304501	NZ_AASH01000000
Geotalea uraniireducens Rf4	5136364	NZ_AAON01000000
Trichlorobacter lovleyi SZ	3994874	NZ_AAVG01000000

Syntrophotalea carbinolica DSM 2380	3665893	NC_007498
Anaeromyxobacter dehalogenans 2CP-1	5029329	NZ_ABKC01000000
Anaeromyxobacter sp. K	5061632	NZ_ABHX01000000
Myxococcus xanthus DK 1622	9139763	NC_008095
Haliangium ochraceum DSM 14365	9446314	NZ_ABTW01000000
Sorangium cellulosum So ce56	13033779	NC_010162
Desulfobacca acetoxidans DSM 11109	3282536	NC_015388
Desulfomonile tiedjei DSM 6799	6527027	NC_018026
Syntrophus aciditrophicus SB	3179300	NC_007759
Syntrophobacter fumaroxidans MPOB	4990251	NZ_AAJF01000000
Aliarcobacter butzleri RM4018	2341251	NC_009850
Campylobacter coli RM2228	1860666	NZ_AAFL01000000
Campylobacter concisus 13826	2099413	NZ_AAQZ01000000
Campylobacter curvus 525.92	1971270	NZ_AARA02000000
Campylobacter fetus subsp. fetus 82-40	1773615	NZ_AANR03000000
Campylobacter hominis ATCC BAA-381	1714951	NC_009713
Campylobacter jejuni RM1221	1777831	NC_003912
Campylobacter lari RM2100	1571661	NZ_AAFK01000000
Campylobacter rectus RM3267	2513107	NZ_ACFU01000000
Campylobacter upsaliensis RM3195	1773834	NZ_AAFJ01000000
Helicobacter acinonychis str. Sheeba	1557588	NC_008230
Helicobacter canadensis MIT 98-5491	1633489	NZ_ABQS01000000
Helicobacter cinaedi CCUG 18818 = ATCC BAA-847	2214470	NZ_ABQT01000000
Helicobacter hepaticus ATCC 51449	1799146	NC_004917
Helicobacter mustelae 12198	1578097	NC_013949
Helicobacter pullorum MIT 98-5489	1951667	NZ_ABQU01000000
Helicobacter pylori 26695	1667892	NC_018939
Wolinella succinogenes DSM 1740	2110355	NC_005090
Sulfurospirillum deleyianum DSM 6946	2306351	NZ_ABUV01000000
Nitratifractor salsuginis DSM 16511	2101285	NC_014935
Sulfurovum sp. NBC37-1	2562277	NC_009663
Sulfuricurvum kujiense DSM 16994	2819357	NC_014763
Sulfurimonas autotrophica DSM 16294	2153198	NC_014506
Sulfurimonas denitrificans DSM 1251	2201561	NZ_AAJA01000000
Caminibacter mediatlanticus TB-2	1663618	NZ_ABCJ01000000
Nitratiruptor sp. SB155-2	1877931	NC_009662
Aeromonas hydrophila subsp. hydrophila ATCC 7966	4744448	NC_008570
Aeromonas salmonicida subsp. salmonicida A449	5040536	NC_009350
Tolumonas auensis DSM 9187	3471292	NZ_ACFZ01000000
Alteromonas macleodii ATCC 27126	4653851	NZ_ABQB01000000
Alteromonas mediterranea DE	4480937	NC_011138

Glaciecola sp. HTCC2999	2560882	NZ_ABST01000000
Marinobacter adhaerens HP15	4651725	NC_017508
Colwellia psychrerythraea 34H	5373180	NC_003910
Ferrimonas balearica DSM 9799	4279159	NC_014541
Idiomarina baltica OS145	2770272	NZ_AAMX01000000
Idiomarina loihiensis L2TR	2839318	NC_006512
Moritella sp. PE36	5223088	NZ_ABCQ01000000
Pseudoalteromonas atlantica T6c	5187005	AAKP01000000
Pseudoalteromonas translucida	3850272	NC_007482
Pseudoalteromonas tunicata D2	4994813	NZ_AAOH01000000
Psychromonas ingrahamii 37	4559598	NZ_AAQS01000000
Psychromonas sp. CNPT3	3052410	NZ_CH902577
Shewanella amazonensis SB2B	4306142	NZ_AAIN01000000
Shewanella baltica OS195	5547544	NZ_AATK01000000
Shewanella benthica KT99	4351589	NZ_ABIC01000000
Shewanella denitrificans OS217	4545906	NZ_AAIU01000000
Shewanella frigidimarina NCIMB 400	4845257	NZ_AAIV01000000
Shewanella halifaxensis HAW-EB4	5226917	NC_010334
Shewanella loihica PV-4	4602594	NZ_AALS01000000
Shewanella oneidensis MR-1	5131424	NC_004347
Shewanella pealeana ATCC 700345	5174581	NZ_AAVJ01000000
Shewanella piezotolerans WP3	5396476	NC_011566
Shewanella putrefaciens 200	4840251	AAWY01000000
Shewanella sediminis HAW-EB3	5517674	NZ_ABDD01000000
Shewanella sp. ANA-3	5251146	NZ_AALH01000000
Shewanella sp. MR-4	4706287	NZ_AALX01000000
Shewanella sp. MR-7	4799109	AALI01000000
Shewanella sp. W3-18-1	4708380	NZ_AALN01000000
Shewanella violacea DSS12	4962103	NC_014012
Shewanella woodyi ATCC 51908	5935403	NZ_AAUO01000000
Alteromonadales bacterium TW-7	4104952	AAVS01000000
Dichelobacter nodosus VCS1703A	1389350	NC_009446
Cellvibrio japonicus Ueda107	4576573	NC_010995
Saccharophagus degradans 2-40	5057531	NZ_AABI03000000
Teredinibacter turnerae T7901	5193164	NC_012997
Congregibacter litoralis KT71	4351117	NZ_AAOA02000000
Allochromatium vinosum DSM 180	3669074	NZ_ACQQ01000000
Nitrosococcus halophilus Nc 4	4145260	NC_013958
Nitrosococcus oceani ATCC 19707	3522111	NC_007483
Alkalilimnicola ehrlichii MLHE-1	3275944	NZ_AALK01000000
Halorhodospira halophila SL1	2678452	NZ_AAOQ01000000

Nitrococcus mobilis Nb-231	3623681	NZ_AAOF01000000
Thioalkalivibrio sp. K90mix	2985056	NZ_ACIU01000000
Thioalkalivibrio sulfidiphilus HL-EbGr7	3464554	NZ_ABYM01000000
Halothiobacillus neapolitanus c2	2582886	NZ_ACJO01000000
Sodalis glossinidius str. 'morsitans'	4292502	NC_007715
Candidatus Hamiltonella defensa 5AT (Acyrthosiphon pisum)	2169363	NC_012752
Citrobacter freundii ATCC 8090 = MTCC 1658 = NBRC 12681	4957773	NZ_CP049015
Citrobacter koseri ATCC BAA-895	4735357	NC_009793
Citrobacter portucalensis	4945242	NZ_CP022311
Citrobacter rodentium ICC168	5444283	NC_013719
Citrobacter youngae ATCC 29220	5154159	NZ_ABWL02000000
Cronobacter sakazakii ATCC BAA-894	4530777	NC_009780
Cronobacter turicensis z3032	4599092	FN543096
Enterobacter asburiae	4625174	NZ_JUGH01000000
Enterobacter cancerogenus ATCC 35316	4638653	NZ_ABWM02000000
Enterobacter cloacae subsp. cloacae ATCC 13047	5598796	NC_014108
Enterobacter sp. 638	4676461	AAVF01000000
Escherichia albertii TW07627	4746590	NZ_ABKX01000000
Escherichia coli str. K-12 substr. MG1655	4641652	NC_000913
Escherichia fergusonii ATCC 35469	4643861	NC_011743
Escherichia sp. 4_1_40B	4911206	NZ_ACDM02000000
Klebsiella sp. M5al	5800138	NZ_CP020657
Klebsiella pneumoniae subsp. pneumoniae MGH 78578	5694894	NC_009653
Klebsiella variicola At-22	5458505	NC_013850
[Enterobacter] lignolyticus SCF1	4814049	NC_014618
Salmonella enterica subsp. enterica serovar Typhimurium str. LT2	4951383	NC_003277
Shigella boydii CDC 3083-94	4874659	NZ_AAKA01000000
Shigella dysenteriae Sd197	4560911	NC_009344
Shigella flexneri 2a str. 2457T	4599354	NC_004741
Shigella sonnei Ss046	5055316	NC_007385
Erwinia amylovora CFBP1430	3833832	NC_013957
Erwinia pyrifoliae Ep1/96	4072846	NC_013263
Erwinia tasmaniensis Et1/99	4067864	NC_010697
Pantoea ananatis LMG 20103	4703373	NC_013956
Pantoea sp. MT58	4675847	NZ_CP061086
Edwardsiella ictaluri 93-146	3812301	NC_012779
Edwardsiella tarda EIB202	3804166	NC_013509
Photorhabdus asymbiotica	5094138	NC_012961
Photorhabdus laumondii subsp. laumondii TTO1	5688987	NC_005126
Proteus mirabilis HI4320	4099895	NC_010555
Providencia alcalifaciens DSM 30120	4029346	NZ_ABXW01000000

Providencia rettgeri DSM 1131	4764068	NZ_ACCI02000000
Providencia rustigianii DSM 4541	3978644	NZ_ABXV02000000
Providencia stuartii ATCC 25827	4610361	NZ_ABJD02000000
Xenorhabdus bovienii SS-2004	4225498	NC_013892
Dickeya chrysanthemi Ech1591	4813854	NC_012912
Dickeya dadantii 3937	4922802	NC_014500
Dickeya dianthicola	4851809	NZ_CP051429
Dickeya parazeae Ech586	4818394	NZ_ACYK01000000
Musicola paradisiaca Ech703	4679450	NC_012880
Pectobacterium atrosepticum SCRI1043	5064019	NC_004547
Pectobacterium carotovorum subsp. carotovorum PC1	4862913	NC_012917
Pectobacterium parmentieri WPP163	5063892	NZ_ACUM01000000
Rahnella sp. WP5	5444112	NZ_JQJR01000000
Serratia liquefaciens	5395544	NZ_CP061082
Serratia proteamaculans 568	5495657	AAUN01000000
Yersinia enterocolitica subsp. enterocolitica 8081	4683620	NC_008791
Yersinia frederiksenii ATCC 33641	4885341	NZ_AALE02000000
Yersinia intermedia ATCC 29909	4711317	NZ_AALF02000000
Yersinia mollaretii ATCC 43969	4549461	NZ_AALD02000000
Yersinia pestis KIM10+	4701745	NC_004838
Yersinia pseudotuberculosis IP 31758	4935125	NZ_AAKT02000000
Kangiella koreensis DSM 16069	2852073	NZ_ABUB01000000
Coxiella burnetii CbuG_Q212	2008870	NC_011527
Rickettsiella grylli	1581239	NZ_AAQJ02000000
Legionella longbeachae NSW150	4149158	NC_014544
Legionella pneumophila str. Corby	3576470	NC_009494
Methylococcus capsulatus str. Bath	3304561	NC_002977
Acinetobacter baumannii AB0057	4063879	NC_011585
Acinetobacter baylyi ADP1	3598621	NC_005966
Acinetobacter sp. ATCC 27244	3434013	NZ_ABYN01000000
Psychrobacter arcticus 273-4	2650701	NZ_AADI01000000
Psychrobacter cryohalolentis K5	3101097	NZ_AAJC01000000
Alcanivorax borkumensis SK2	3120143	NC_008260
Alcanivorax jadensis T9	3629371	NZ_ARXU01000000
Hahella chejuensis KCTC 2396	7215267	NC_007645
Chromohalobacter salexigens DSM 3043	3696649	NZ_AAHZ01000000
Kangiella aquimarina DSM 16071	2681803	NZ_ARFE01000000
Bermanella marisrubri	3552236	NZ_AAQH01000000
Marinomonas sp. MED121	5153226	NZ_AANE01000000
Marinomonas sp. MWYL1	5100344	AAVH01000000
Neptuniibacter caesariensis	3924755	NZ_AAOW01000000

Reinekea blandensis MED297	4526761	NZ_AAOE01000000
Actinobacillus minor 202	2127148	NZ_ACFT01000000
Actinobacillus pleuropneumoniae serovar 1 str. 4074	2292348	NZ_AACK01000000
Actinobacillus succinogenes 130Z	2319663	AAKC01000000
Aggregatibacter actinomycetemcomitans D11S-1	2160722	NC_014629
Aggregatibacter aphrophilus NJ8700	2313035	NC_012913
[Mannheimia] succiniciproducens MBEL55E	2314078	NC_006300
Glaesserella parasuis SH0165	2269156	NC_011852
[Haemophilus] ducreyi 35000HP	1698955	NC_002940
Haemophilus influenzae 86-028NP	1914490	NZ_AAET01000000
Histophilus somni 2336	2263857	AACJ01000000
Pasteurella multocida subsp. multocida str. Pm70	2257487	NC_002663
Marinobacter algicola DG893	4413003	NZ_ABCP01000000
Marinobacter halophilus	3917476	NZ_PXNN01000000
Marinobacter sp. ELB17	4894744	NZ_AAXY01000000
Psychrobacter sp. PRwf-1	2995049	AAPX01000000
Azotobacter vinelandii DJ	5365318	NZ_AAAU03000000
Pseudomonas aeruginosa PAO1	6264404	NC_002516
Pseudomonas coronafaciens pv. oryzae str. 1_6	5714458	NZ_CP046036
Pseudomonas entomophila L48	5888780	NC_008027
Pseudomonas fluorescens Pf0-1	6438405	NZ_AAAT03000000
Pseudomonas mendocina ymp	5072807	AAUL01000000
Pseudomonas protegens Pf-5	7074893	NC_004129
Pseudomonas putida KT2440	6181873	NC_002947
Pseudomonas savastanoi pv. phaseolicola 1448A	6112448	NZ_AAEZ01000000
Pseudomonas simiae	6169071	NZ_CP007637
Pseudomonas stutzeri RCH2	4600489	NC_019939
Pseudomonas syringae pv. syringae B728a	6093698	NZ_AABP02000000
Candidatus Ruthia magnifica str. Cm (Calyptogena magnifica)	1160782	NC_008610
Francisella tularensis subsp. mediasiatica FSC147	1893886	CP000915
Hydrogenovibrio crunogenus XCL-2	2427734	CP000109
Methylophaga thiooxydans DMS010	3047652	NZ_ABXT01000000
Thiothrix nivea DSM 5205	4691711	NZ_AJUL01000000
Aliivibrio fischeri ES114	4273718	NC_006842
Aliivibrio salmonicida LFI1238	4655660	NC_011311
Photobacterium angustum S14	5181543	NZ_AAOJ01000000
Photobacterium profundum SS9	6403280	NC_005871
Photobacterium sp. SKA34	4992772	NZ_AAOU01000000
Vibrio alginolyticus 12G01	5257379	NZ_AAPS01000000
Vibrio antiquarius	5089025	NC_013457
Vibrio atlanticus	4974818	NC_011744

Vibrio campbellii ATCC BAA-1116	6058377	NC_009777
Vibrio cholerae O1 biovar El Tor	4082461	NZ_CP047304
Vibrio parahaemolyticus RIMD 2210633	5165770	NC_004605
Vibrio shilonii AK1	5701826	NZ_ABCH01000000
Vibrio sp. 16	4535833	NZ_ACCV01000000
Vibrio sp. AND4	4255798	NZ_ABGR01000000
Vibrio sp. MED222	4984034	NZ_AAND01000000
Vibrio splendidus 12B01	5702234	NZ_AAMR01000000
Vibrio vulnificus CMCP6	5126696	NC_004459
Vibrionales bacterium SWAT-3	5835713	AAZW01000000
Dyella japonica UNC79MFTsu3.2	5018507	NZ_JIBD01000000
Rhodanobacter denitrificans	3959115	NZ_CP088922
Rhodanobacter sp. FW104-R8	3722033	NZ_LVJR01000000
Rhodanobacter sp. FW510-R10	3900803	NZ_LVJU01000000
Rhodanobacter sp. FW510-T8	3722824	NZ_LVJT01000000
Rhodanobacter thiooxydans	4203948	NZ_CP088924
Lysobacter sp. OAE881	4203097	JADBDJ010000000
Stenotrophomonas maltophilia K279a	4851126	NC_010943
Stenotrophomonas sp. SKA14	5020454	NZ_ACDV01000000
Xanthomonas albilineans	3852299	NC_017555
Xanthomonas campestris pv. campestris str. 8004	5148708	NC_007086
Xanthomonas citri pv. citri str. 306	5274174	NC_003922
Xanthomonas oryzae pv. oryzae MAFF 311018	4940217	NC_007705
Xylella fastidiosa M12	2475130	NC_010513
Bdellovibrio bacteriovorus HD100	3782950	NC_005363
Mariprofundus ferrooxydans PV-1	2992730	NZ_AATS01000000
Brachyspira hyodysenteriae WA1	3036634	NC_012226
Leptospira biflexa serovar Patoc strain 'Patoc 1 (Ames)'	3956089	NC_010846
Leptospira borgpetersenii serovar Hardjo-bovis str. JB197	3876235	CP000351
Leptospira interrogans serovar Copenhageni str. Fiocruz L1-130	4627366	NC_005824
Borrelia duttonii Ly	1574881	CP000979
Borrelia recurrentis A1	1242163	NC_011246
Borrelia turicatae 91E135	1165365	NZ_CP019369
Borreliella afzelii ACA-1	1353819	NZ_ABCU02000000
Borreliella burgdorferi 118a	1453013	NZ_ABGI02000000
Borreliella finlandensis	1281782	NZ_ABJZ02000000
Borreliella garinii PBr	1265591	NZ_ABJV02000000
Borreliella spielmanii A14S	1252559	NZ_ABKB02000000
Borreliella valaisiana VS116	1258864	NC_012177
Gracilinema caldarium DSM 7334	3239340	NC_015732
Sphaerochaeta coccoides DSM 17374	2227296	NC_015436

Spirochaeta africana DSM 8902	3285855	NZ_AGRZ01000000
Spirochaeta thermophila DSM 6578	2560222	NZ_AEOG01000000
Treponema brennaborense DSM 12168	3055580	NC_015500
Treponema denticola ATCC 35405	2843201	NC_002967
Treponema pallidum subsp. pallidum SS14	1139457	NC_010741
Treponema saccharophilum DSM 2985	3453898	NZ_AGRW01000000
Treponema succinifaciens DSM 2489	2897425	NC_015386
Aminobacterium colombiense DSM 12261	1980592	NC_014011
Aminomonas paucivorans DSM 12260	2630170	NZ_AEIV01000000
Cloacibacillus evryensis DSM 19522	3488464	NZ_JFBR01000000
Thermanaerovibrio acidaminovorans DSM 6589	1848474	NZ_ABUW01000000
Thermovirga lienii DSM 17291	1999646	NC_016149
Nitrosopumilus maritimus SCM1	1645259	NZ_ABEO01000000
Thermodesulfatator indicus DSM 15286	2322224	NC_015681
Kosmotoga olearia TBF 19.5.1	2302126	NZ_ABZD01000000
Petrotoga mobilis SJ95	2169548	NZ_AAZB01000000
Fervidobacterium nodosum Rt17-B1	1948941	NZ_AAUK01000000
Thermosipho africanus TCF52B	2016657	NC_011653
Thermosipho melanesiensis BI429	1915238	NZ_AAWH01000000
Pseudothermotoga lettingae TMO	2135342	NC_009828
Pseudothermotoga thermarum DSM 5069	2039943	NC_015707
Thermotoga maritima MSB8	1860725	NC_000853
Thermotoga naphthophila RKU-10	1809823	NZ_ACXW01000000
Thermotoga neapolitana DSM 4359	1884562	NC_011978
Thermotoga petrophila RKU-1	1823511	NZ_AAWB01000000
Thermotoga sp. RQ2	1877693	NZ_ABIJ01000000
Methylacidiphilum infernorum V4	2287145	NC_010794
Opitutus terrae PB90-1	5957605	NC_010571
Coraliomargarita akajimensis DSM 45221	3750771	NC_014008
Chthoniobacter flavus Ellin428	7848700	NZ_ABVL01000000
Akkermansia muciniphila ATCC BAA-835	2664102	NZ_ABJN01000000
Verrucomicrobium spinosum DSM 4136 = JCM 18804	8220857	NZ_ABIZ01000000
Verrucomicrobiae bacterium DG1235	5775745	ABSI01000000
